# Design of Wide-Spectrum Inhibitors Targeting Coronavirus Main Proteases

**DOI:** 10.1371/journal.pbio.0030324

**Published:** 2005-09-06

**Authors:** Haitao Yang, Weiqing Xie, Xiaoyu Xue, Kailin Yang, Jing Ma, Wenxue Liang, Qi Zhao, Zhe Zhou, Duanqing Pei, John Ziebuhr, Rolf Hilgenfeld, Kwok Yung Yuen, Luet Wong, Guangxia Gao, Saijuan Chen, Zhu Chen, Dawei Ma, Mark Bartlam, Zihe Rao

**Affiliations:** **1**Tsinghua-IBP Joint Research Group for Structural Biology, Tsinghua University, Beijing, China,; **2**National Laboratory of Biomacromolecules, Institute of Biophysics, Chinese Academy of Sciences, Beijing, China,; **3**State Key Laboratory of Bioorganic and Natural Products Chemistry, Shanghai Institute of Organic Chemistry, Chinese Academy of Sciences, Shanghai, China,; **4**Shanghai Institute of Hematology, Rui-Jin Hospital affiliated to Shanghai Second Medical University, Shanghai, China,; **5**Guangzhou Institute of Biomedicine and Health, Chinese Academy of Sciences, Guangzhou, China,; **6**Institute of Virology and Immunology, University of Würzburg, Würzburg, Germany,; **7**Institute for Biochemistry, University of Lübeck, Lübeck, Germany,; **8**Department of Microbiology, University of Hong Kong, Hong Kong, China,; **9**Department of Chemistry, Inorganic Chemistry Laboratory, University of Oxford, Oxford, United Kingdom; Howard Hughes Medical Institute/California Institute of TechnologyUnited States of America

## Abstract

The genus *Coronavirus* contains about 25 species of coronaviruses (CoVs), which are important pathogens causing highly prevalent diseases and often severe or fatal in humans and animals. No licensed specific drugs are available to prevent their infection. Different host receptors for cellular entry, poorly conserved structural proteins (antigens), and the high mutation and recombination rates of CoVs pose a significant problem in the development of wide-spectrum anti-CoV drugs and vaccines. CoV main proteases (M^pro^s), which are key enzymes in viral gene expression and replication, were revealed to share a highly conservative substrate-recognition pocket by comparison of four crystal structures and a homology model representing all three genetic clusters of the genus *Coronavirus.* This conclusion was further supported by enzyme activity assays. Mechanism-based irreversible inhibitors were designed, based on this conserved structural region, and a uniform inhibition mechanism was elucidated from the structures of M^pro^-inhibitor complexes from severe acute respiratory syndrome-CoV and porcine transmissible gastroenteritis virus. A structure-assisted optimization program has yielded compounds with fast in vitro inactivation of multiple CoV M^pro^s, potent antiviral activity, and extremely low cellular toxicity in cell-based assays. Further modification could rapidly lead to the discovery of a single agent with clinical potential against existing and possible future emerging CoV-related diseases.

## Abstract 2

Structure-assisted optimization of compounds capable of inactivating CoV M^pro^s may rapidly lead to an antiviral agent against *Coronavirus*-associated diseases.

## Introduction

The genus *Coronavirus* belongs to the plus-strand RNA virus family of the *Coronaviridae* and currently contains about 25 species that are classified into three groups according to their genetic and serological relationships [1–4]. Coronaviruses (CoVs) infect humans and multiple species of animals, causing a variety of highly prevalent and severe diseases [1,5]. For example, human coronavirus (HCoV) strains 229E (HCoV-229E), NL63 (HCoV-NL63), OC43 (HCoV-OC43), and HKU1 (HCoV-HKU1) cause a significant portion of upper and lower respiratory tract infections in humans, including common colds, bronchiolitis, and pneumonia. They have also been implicated in otitis media, exacerbations of asthma, diarrhea, myocarditis, and neurological disease [2,3,6–9]. A previously unknown HCoV, severe acute respiratory syndrome coronavirus (SARS-CoV), which is most closely related to the group II CoVs [10], proved to be the etiological agent of a global outbreak of a life-threatening form of pneumonia called severe acute respiratory syndrome (SARS), which, in 2003, was the cause of more than 800 fatalities worldwide [11–14]. Animal CoVs are mainly associated with enteric and respiratory diseases in livestock and domestic animals. Most of the viruses are highly contagious with significant mortality in young animals, resulting in considerable economic losses worldwide [5,9].

Although vaccines have been developed against avian infectious bronchitis virus (IBV), canine CoV, and porcine transmissible gastroenteritis virus (TGEV) to help prevent serious diseases, several potential problems remain. Vaccination against IBV is only partially successful due to the continual emergence of new serotypes and recombination events between field and vaccine strains. The development of vaccines against feline infectious peritonitis virus (FIPV) has been frustrated by the phenomenon of antibody-dependent enhancement. No licensed vaccines or specific drugs are available to prevent HCoV infection [6,9]. Following the SARS outbreak, a series of inhibitors was reported against the helicase and main protease (M^pro^) of SARS-CoV to prevent viral replication [15–20]. However, previous research has only placed emphasis on SARS-CoV, and no structural data are available to confirm the direct interaction between these inhibitors and their targets, or for the further modification of these compounds.

In common with other RNA viruses employing RNA-dependent RNA polymerases for genome replication, CoVs are generally thought to mutate at a high frequency [21], although this phenomenon remains to be studied in detail. During the SARS epidemic in China, the emergence of SARS-CoV suggested an animal–human interspecies transmission [22,23]. The virus continued evolving to adapt to the human host during the course of the outbreak [22] with about one-third the mutation rate of human immunodeficiency virus [24]. The high degree of similarity between genome sequences of bovine CoV and the recently sequenced HCoV-OC43 suggested an earlier animal-to-human interspecies transmission than SARS-CoV [25]. Moreover, a high frequency of RNA recombination is a common feature of CoV genetics and has been demonstrated for representative viruses from all CoV groups, including murine hepatitis virus (MHV), TGEV, and IBV [9,26]. For instance, the outbreaks caused by variant strains of IBV that arose from recombination of vaccine and wild-type virulent strains in chicken flocks limit the usage of vaccines against IBV [27,28]. Consequently, it is of concern whether current vaccines or drugs in development will be effective against the next wave of attacks by altered SARS-CoV [22].

In view of the issues posed above, the development of wide-spectrum drugs against the existing pathogenic CoVs is a more reasonable and attractive prospect than individual strategies for drug design, and thereby could provide an effective first line of defense against future emerging CoV-related diseases such as SARS. However, some of the key factors controlling the host spectrum and viral pathogenicity are highly variable among CoVs. For instance, CoVs use different host receptors for cellular entry, have poorly conserved structural proteins (antigens), and encode diverse accessory genes in their 3'-terminal genome regions that probably contribute to the pathogenicity of CoVs in specific hosts [1–3,29–34]. Clearly, this structural and functional diversity presents a significant obstacle for designing a versatile compound against all CoVs unless a highly conserved target that is comparatively stable during evolution is identified within the genus *Coronavirus.* Here we report the discovery of a highly conserved region based on four crystal structures and one homology model of M^pro^ representing all three genetic clusters of the genus *Coronavirus,* and a uniform inhibition mechanism revealed from the structures of M^pro^-inhibitor complexes from SARS-CoV and TGEV. A structure-assisted optimization program has yielded compounds with fast in vitro inactivation of multiple CoV M^pro^s, potent antiviral activity, and extremely low cellular toxicity in cell-based assays. Further modification could rapidly lead to the discovery of a single agent with clinical potential against existing and possible future emerging CoV-associated diseases.

## Results/Discussion

### Target Identification

Development of wide-spectrum inhibitors is an attractive strategy against CoV-associated diseases; however, it entirely depends on the availability of a conserved target within the whole genus *Coronavirus.* During the first round of target screening, all structural proteins (including S, E, M, HE, and N proteins) were excluded due to the considerable variations among different CoVs [1–3,33,34]. Subsequently, the RNA-dependent RNA polymerase, RNA helicase, and M^pro^ constitute attractive potential nonstructural protein targets for consideration. However, no structural data were available for the former two proteins, increasing the difficulties for rational drug design and downstream modification of possible drug leads.

The pivotal roles played by M^pro^s in controlling viral replication and transcription through extensive processing of replicase polyproteins, together with the absence of closely related cellular homologues, identify the M^pro^ as a potentially important target for antiviral drug design [35]. However, pairwise BLAST of the primary sequences among CoV M^pro^s showed identities of only 38% in some cases. Since it is acknowledged that three-dimensional structures are more closely conserved than primary sequences, we decided to investigate the conservation among the CoV M^pro^ structures. As the M^pro^s showed comparatively high sequence similarity within each CoV group, representatives from every group were chosen for comparison. The structures of M^pro^s from TGEV (group I), HCoV-229E (group I), and SARS-CoV are available [36–38]. Although the crystal structure of IBV (group III) M^pro^ is currently under refinement by our group, it can nevertheless be used as an experiment-based model. As the structure of MHV M^pro^ (group II) was unavailable, and previous studies have shown that SARS-CoV is related to group II [10], we constructed a homology model for MHV M^pro^ based on the structure of SARS-CoV M^pro^. Superposition of the crystal structures and homology model showed approximately 2 Å root mean square deviation for all 300 C_α_, but the most variable regions were the helical domain III and surface loops. The substrate-binding pockets located in a cleft between domains I and II, and especially the S4, S2, and S1 are highly conserved among CoV M^pro^s suggesting the possibility for wide-spectrum inhibitor design targeting this region in the M^pro^s of all CoVs. This hypothesis was further supported by enzyme activity assays (see Table 1). Based on the assumption that the substrate-binding sites are highly conserved among CoV M^pro^s, a fluorescence-labeled substrate MCA-AVLQ↓SGFR-Lys(Dnp)-Lys-NH2 was synthesized to determine the kinetic parameters of TGEV, HCoV-229E, FIPV, MHV, IBV, and SARS-CoV M^pro^s. The substrate sequence was derived from residues P4–P5′ of the SARS-CoV M^pro^ N-terminal autoprocessing site, which has the sequence AVLQSGFRK. IBV M^pro^ demonstrated an almost identical *K_m_* to that of SARS-CoV M^pro^. An interesting observation was that four other CoV proteases showed marginally stronger binding affinity to the substrate than SARS-CoV M^pro^ itself. These results further support the preliminary biochemical studies on conservation of substrates of CoV M^pro^s [39].

**Table 1:**
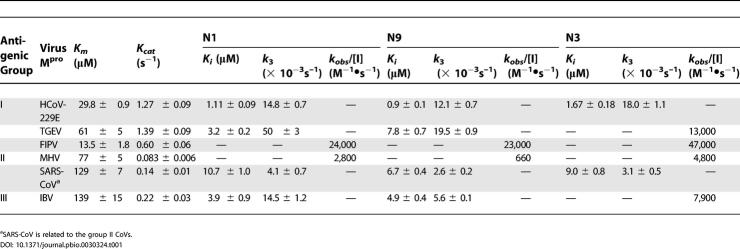
Enzyme Activity and Enzyme-Inhibition Data for Representatives of All Genetic Clusters of Genus *Coronavirus*

### First Round of Inhibitor Design: Michael Acceptor Inhibitors

The structures of TGEV and SARS-CoV M^pro^s have previously been determined in complex with a substrate-analog chloromethyl ketone (CMK) inhibitor, Cbz-VNSTLQ-CMK. The sequence of this substrate-analog was derived from residues P6–P1 of the N-terminal autoprocessing site of TGEV M^pro^ [36,38]. However, the two protomers of SARS-CoV M^pro^ each exhibited an unexpected binding mode, possibly resulting from the comparatively weak binding of peptidyl elements derived from the substrate of TGEV M^pro^ and from the highly reactive electrophile CMK. This would suggest that nucleophilic attack might have occurred before a stable noncovalently bound enzyme-inhibitor complex was formed. Accordingly, the single binding mode in the TGEV M^pro^ complex was taken into account when designing possible broad-spectrum inhibitors on the basis of these structures and models. Although the CMK inhibitor is nonselective because of its high chemical reactivity and is susceptible to cleavage by gastric and enteric proteinases, it could provide structural insight into the substrate-binding pocket. Superposition of the structures and model revealed that all these proteases have a His–Cys catalytic dyad with relatively conserved orientations, in which His acts as a proton acceptor and Cys undergoes nucleophilic attack on the carbonyl carbon of the substrate. It is widely accepted that increased inhibitor potency can be achieved provided that a covalent bond is formed between the active Cys residue and the designed compound, resembling the intermediate during substrate cleavage. The Michael acceptors, a class of conjugated carbonyl compounds, were successfully introduced to devise irreversible Cys protease inhibitors, including the antirhinovirus compound ruprintrivir (formerly designated AG7088) [40–42], and so the highly reactive electrophile CMK was replaced by a less reactive *trans*-α, β-unsaturated ethyl ester, which was expected to readily extend into the bulky S1′ subsite of CoV M^pro^s.

**Figure 1:**
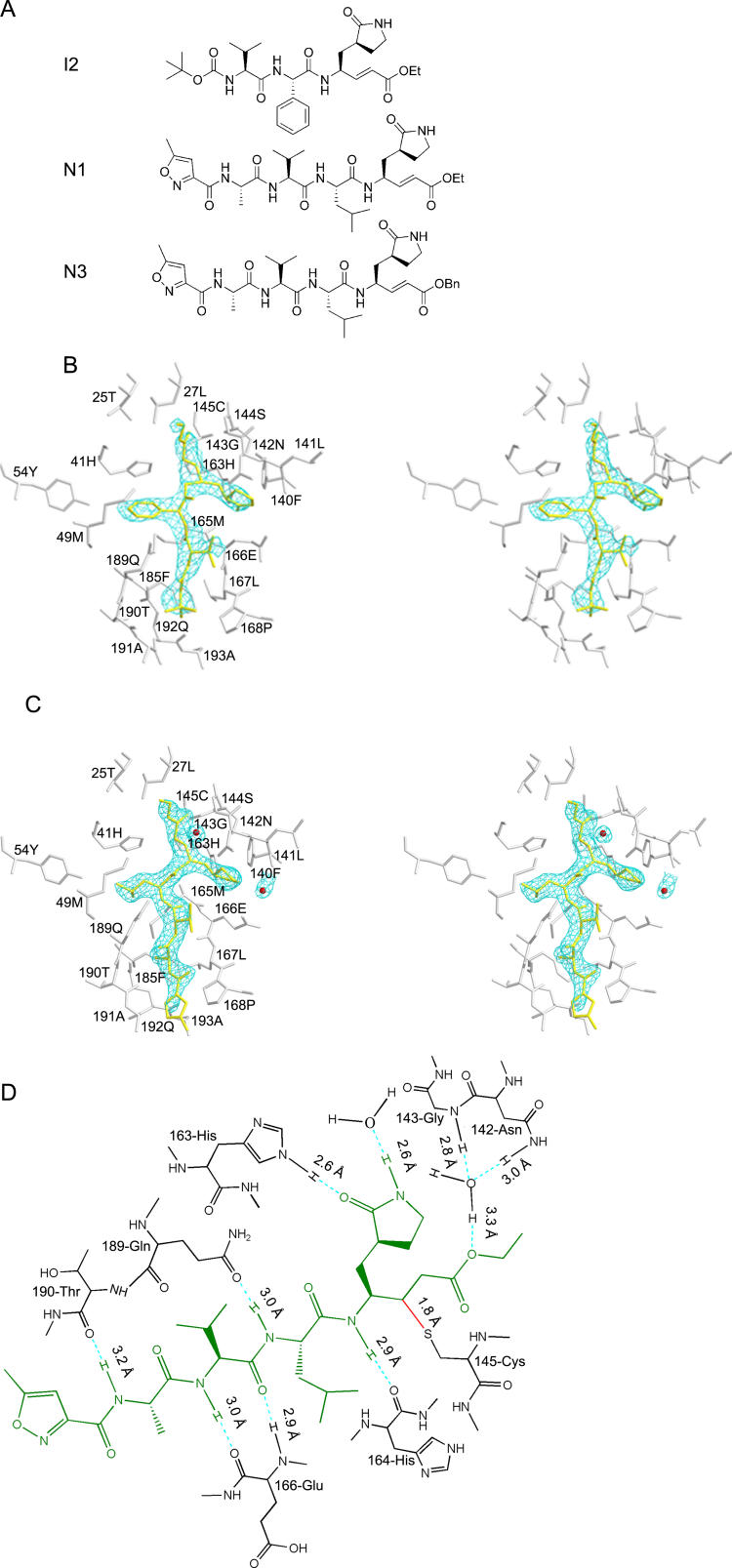
Structures of Inhibitors and Their Interactions with SARS-CoV M^pro^ (A) The structures of compounds I2, N1, and N3. (B) A stereo view showing I2 bound into the substrate-binding pocket of the SARS-CoV M^pro^ at 2.7 Å. The I2 inhibitor is shown in gold and covered by an omit map contoured at 1.0 σ. Residues forming the substrate-binding pocket are shown in silver. (C) A stereo view showing N1 bound into the substrate-binding pocket of the SARS-CoV M^pro^ at 2.0 Å. The N1 inhibitor is shown in gold and covered by an omit map contoured at 1.0 σ. Residues forming the substrate-binding pocket are shown in silver. Two water molecules (in red) form hydrogen bonds with N1. (D) Detailed view of the interactions between the N1 and SARS-CoV M^pro^. The N1 inhibitor is shown in green. Hydrogen bonds are shown as dashed lines, and interaction distances are given. The covalent bond is labeled in red.

During our initial round of inhibitor design, we focused on the S1, S2, and S4 subsites crucial for substrate recognition and utilized a strategy for mimicking the substrate side chains of residues P4–P1 to accommodate the corresponding subsites. Since backbones of CoV M^pro^s constituting this area superimposed particularly well, except for a small segment located on the outer wall of S2, we concentrated on the variation of side chains forming these pockets. In the TGEV M^pro^ complex structure [36], the side chains of 165-Glu, 162-His, 171-His, and 139-Phe (also conserved in other M^pro^s) are incorporated with other backbone elements to constitute the S1 site, which has an absolute requirement for Gln at the P1 position via two hydrogen bonds. Modeling showed that a lactam with (S) stereochemistry at the α-carbon might preserve the hydrogen bonds essential for S1 recognition; moreover, a comparatively bulky lactam ring would create additional van der Waals interactions. The side chains of 164-Leu, 51-Ile, 41-His, and 53-Tyr, as well as the alkyl portion of side chains of 186-Asp and 47-Thr, are involved in forming a deep hydrophobic S2 subsite that can accommodate the relatively large side chain of Leu in TGEV M^pro^. This feature can also be observed in the HCoV-229E M^pro^. Several conservative substitutions occur in other CoV M^pro^s (164-Leu → 165-Met in SARS-CoV and MHV M^pro^s; 53-Tyr → 50-Trp in IBV M^pro^). Another minor difference was observed in SARS-CoV and MHV M^pro^s, where the outer wall segment is composed of a short 3_10_-helix from residues 45–50, compared with a less regular structure in HCoV and TGEV M^pro^. With respect to the structure of IBV M^pro^ undergoing refinement, no clear electron density was observed in the corresponding stretch of residues 44–47. We reasoned that variations in the segment located on the outer wall of S2 should not significantly affect the hydrophobicity of this deep subsite. This is supported by evidence wherein Leu is found at position P2 of substrates for all CoV M^pro^s. As P2 Phe is present in the C-terminal autocleavage site of SARS-CoV, phenyl was used as a smaller substituent to explore this subsite. The side chain of Thr at P3 is solvent-exposed, so this site was expected to tolerate a wide range of functionality. The side chains of 164-Leu, 166-Leu, 184-Tyr, and 191-Gln that form the S4 hydrophobic subsite of TGEV are conserved in other CoV M^pro^s, excluding the following conservative substitutions: 184-Tyr → 184-Phe in HCoV M^pro^; 164-Leu → 165-Met, 184-Tyr → 185-Phe in SARS-CoV. A tertiary butyloxycarbonyl was introduced at the P4 position as an N-terminal protecting group to enter into the S4 site. Thus, by combining the modifications above, a novel compound designated as I2 (see Figure 1A) was designed and a series of analogs was synthesized for the inhibition assay (see Protocol S1).

### Kinetic Mechanism of Michael Acceptor Inhibitors

Covalent irreversible inactivation of CoV M^pro^s by Michael acceptor inhibitors proceeds according to the kinetic mechanism presented in the scheme below:



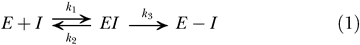



The inhibitor initially forms a reversible complex with the protease, which then undergoes a chemical step (nucleophilic attack by Cys) leading to the formation of a stable covalent bond [42]. The evaluation of this series of time-dependent inhibitors requires both the equilibrium-binding constant *K_i_* (designated as *k*_2_/*k*_1_) and the inactivation rate constant for covalent bond formation *k*_3_ [43]. We avoided measurement of IC_50_ after preincubation to assess the effect of these time-dependent inhibitors, since there is a general trend for this value to decrease to zero with prolonged preincubation time, which would lead to an inappropriate evaluation.

### The Structure of SARS-CoV M^pro^ in Complex with an Inhibitor I2

The compounds designed in the first round did not exhibit obvious inhibition on CoV M^pro^s without preincubation, suggesting a very poor *K_i_.* We were able to solve a 2.7-Å resolution crystal structure of SARS-CoV M^pro^ complexed with I2 (see Figure 1B; Table S1) despite the weak noncovalent binding, in order to enhance the inhibitory effect of these compounds. Briefly, compound I2 binds to the shallow cleft formed by a portion of the strand eII and a segment of the loop linking domains II and III. The C_β_ atom of the Michael acceptor forms a covalent bond with Sγ of 145-Cys as expected. The lactam P1 inserts favorably into S1 and the side chain of Val at P3 is solvent-exposed. However, the failure of P2 and P4 to be properly accommodated by their corresponding subsites attracted our attention, and might account for the poor inhibitory effect of this series of molecules. First, although phenyl at P2 could enter the S2 site, its rigidity prevents it from reorienting to insert further into this site. Second, the N-terminal protecting group tertiary butyloxycarbonyl did not insert into the S4 subsite, possibly as a result of the planar property of the butyloxyamide group. The other compounds designed in this round are listed in Table S2.

### Second Round of Inhibitor Design: Optimization of Michael Acceptor Inhibitors

Based on the complex structure of I2, we entered into a second round of optimization focusing on the P2 and P4 recognition sites. For the P2 subsite, the phenyl group was substituted by a more flexible Leu side chain. In order to enhance the binding affinity, a series of residues were utilized as substituents at P4, followed by a heterocycle that should increase the Van der Waals contacts with residues flanking at either side. From this round of modification, two inhibitors designated as N1 and N9, and a more efficacious derivative named N3, were identified with fast in vitro inactivation of all CoV M^pro^s tested, including those of TGEV, HCoV-229E, FIPV, HCoV-NL63 (representatives from group I); MHV, HCoV-HKU1 (representatives from group II); SARS-CoV (related to group II); and IBV (representative from group III) in preliminary inhibition assays (see Figure S2). These inhibitors are not sensitive under 1 mM concentration of dithiothreitol (DTT), which is consistent with a previous report of this type of compound [42]. Subsequently, strict inhibition kinetic parameters were determined and are listed in Table 1 (determination of kinetic parameters of M^pro^s of HCoV-HKU1 and HCoV-NL63 is underway). These inhibitors showed more powerful inhibition of FIPV M^pro^ than other proteases with high inactivation rates (*k_obs_*/[I] ≥ 23,000 M^−1^•s^−1^), such that measurement of *K_i_* and *k*_3_ proved difficult. In this case, *k_obs_*/[I] was utilized to evaluate their inhibition as an approximation of the pseudo second-order rate constant (*k*_3_/*K_i_*) if very rapid inactivation occurs. The *K_i_* of N1 ranges from approximately 1.11–10.7 μM and *k*_3_ ranges from approximately 4.1–50 × 10^−3^s^−1^; the *K_i_* of N9 ranges from approximately 0.9–6.7 μM, and *k*_3_ ranges from approximately 2.6–19.5 × 10^−3^s^−1^. Compared with N1 and N9, N3 demonstrated more potent inhibition on TGEV, FIPV, MHV, and IBV M^pro^s with *k_obs_*/[I] ranging from approximately 4,700–47,000 M^−1^•s^−1^. We therefore solved the crystal structure of SARS-CoV and TGEV M^pro^s individually complexed with N1, which revealed a common mechanism of inhibition among CoV M^pro^s.

### The Structure of SARS-CoV M^pro^ in Complex with the Inhibitor N1

**Figure 2:**
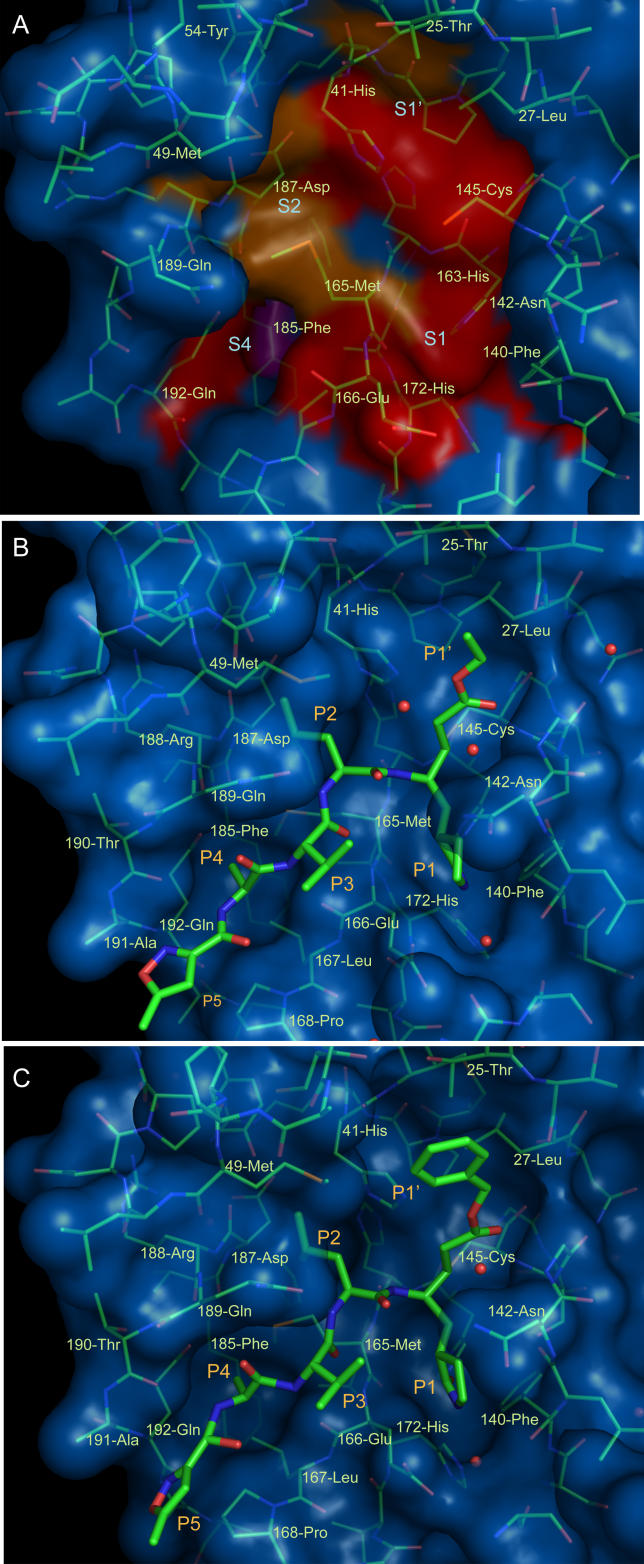
Surface Representation of Native SARS-CoV M^pro^ and Inhibitor Complexes (A) Surface representation of conserved substrate-binding pockets of five CoV M^pro^s. Background is SARS-CoV M^pro^. Red: identical residues among the five CoV M^pro^s; magenta: substitution in one CoV M^pro^; orange: substitution in two CoV M^pro^s. The S1, S2, S4, and S1′ subsites and residues forming the substrate-binding pocket are labeled. (B) Surface representation of SARS-CoV M^pro^ (blue) complexed with N1 (green). Water molecules are shown as red spheres. The P1–P5 and P1′ groups and residues forming the substrate-binding pocket are labeled. (C) Surface representation of SARS-CoV M^pro^ (blue) in complex with N3 (green). Labels are the same as in Figure 2B.

N1 binds to protomers A and B of SARS-CoV in an identical and normal manner. On binding N1, the S1 subsite in protomer B adopts an active conformation compared with the partially collapsed S1 pocket of protomer B in the native structure [38], which can be ascribed to inhibitor-induced conformational changes. As a result, discussion will be focused entirely on protomer A (see Figures 1C, 1D, 2A, and 2B). From the omit map (contoured at 1.2 σ), clear electron density showed that N1 binds in an extended conformation with the inhibitor backbone atoms forming an antiparallel sheet with residues 164–168 of the long strand eII on one side, and with residues 189–191 of the loop linking domains II and III. Here we dissect the inhibitor into different parts for further discussion.

#### Gate-regulated switch

Comparison between the molecular surfaces of SARS-CoV M^pro^ complexed with N1 and the native enzyme show that certain residues constituting the S1 and S2 subsites undergo large conformational changes on inhibitor binding (see Figure 2A and 2B). The side chain of 142-Asn flips over with a 6-Å shift to superpose onto the lactam like a lid when P1 inserts into the subsite. This might account for the movement of main chains of residues 141–143 toward the S1 site; 142-Asn, together with the main chains of neighboring residues, covers the P1 site like one half of a gate. On the opposite side, 49-Met protrudes by around 5Å from the hydrophobic S2 site and is situated parallel to the side chain of Leu at P2. The side chain of another residue, 189-Gln, moves upward to form a 3.0-Å hydrogen bond with the backbone NH of P2. These two residues constitute the other half of a gate. Together, these two halves should serve as a gate-regulated switch with an essential role in substrate or inhibitor recognition and binding.

#### *Trans*-α, β-unsaturated ethyl ester

Clear electron density showed that the S_γ_ atom of 145-Cys forms a standard 1.8-Å C–S covalent bond with the C_β_ of vinyl group, which suggests a Michael addition reaction. The S_γ_ atom moved slightly (approximately 0.6 Å) toward the interior of the protein compared with the native enzyme. The Michael acceptor remains in a plane following the Michael addition since it is stabilized by a water molecule. This ordered water molecule donates a long 3.3-Å hydrogen bond to the carboxylate oxygen of the ester and then accepts a 2.8-Å hydrogen bond from the backbone NH of 143-Gly and a 3.0-Å hydrogen bond from the carboxamide nitrogen of 142-Asn. The position of S_γ_ in 145-Cys implies that it undergoes nucleophilic attack on C_β_ by approaching the π-electron cloud from above. The carbonyl oxygen occupies the oxyanion hole and is close to backbone NHs of 143-Cys and 145-Cys, mimicking the tetrahedral oxyanion intermediate formed during Ser protease cleavage. However, the standard hydrogen bonds are not formed. The ethyl ester portion extends into the S1′ site, with sufficient size, and in an extended conformation, to interact with the alkyl portions of 25-Thr and 27-Leu by van der Waals interaction.

#### P1, P2, and P4 sites

The lactam at P1 inserts favorably into the S1 subsite and forms two stable 2.6-Å hydrogen bonds: one between the lactam oxygen and NE2 of 163-His, and another between the lactam NH and a water molecule at the bottom of this subsite. The C_α_ of Leu at the P2 site in N1 moves into the S2 subsite by approximately 1 Å relative to the corresponding carbon atom in I2, and C_β_–C_γ_ of Leu forms an angle of approximately 40° to the phenyl at P2 in the I2 complex, inserting deeply into the S2 subsite. Another notable difference between N1 and I2 is the insertion of an Ala between P3 and P4 in I2, the latter of which was replaced by an isoxazole to block the N-terminal. As expected, the side chain of Ala at the current P4 position readily enters into the S4 subsite. Simultaneously, the backbone NH of Ala donates a hydrogen bond to the carbonyl oxygen of 190-Thr. The isoxazole at P5 makes Van der Waals contacts with 168-Pro and the backbone of residues 190–191.

#### Further modifications of N1

A variety of substitutions were investigated for P4, P5, and P1′ (see Table S3). The 1.85-Å crystal structure of SARS-CoV M^pro^ complexed with N9 (see Figure S1) showed that Val could serve as a substituent at P4, slightly increasing the hydrophobic interactions. Another derivative N3 with benzyl ester exhibited improved inhibition, which could be seen from inhibition assays of FIPV and MHV M^pro^s (see Table 1). Its co-crystal structure with SARS-CoV M^pro^ indicated that the bulky benzyl group extends into the S1′ site, possibly enhancing the Van der Waals interaction with 25-Thr and 27-Leu (see Figure 2C).

### The Structure of TGEV M^pro^ in Complex with an Inhibitor N1

**Figure 3:**
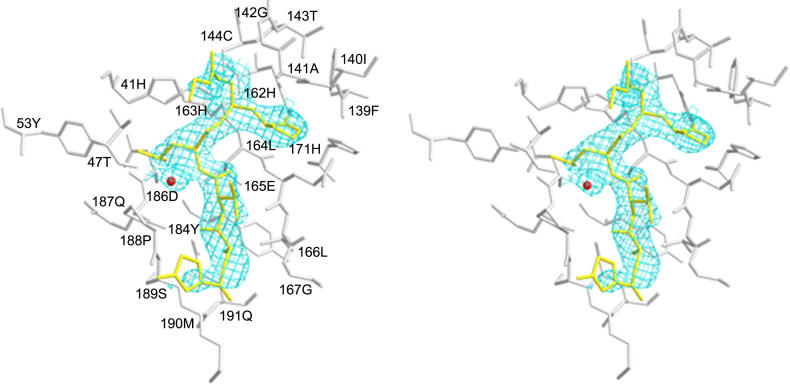
The Structure of TGEV M^pro^ in Complex with N1 A stereo view showing N1 bound into the substrate-binding pocket of the TGEV M^pro^ at 2.7 Å. The N1 inhibitor is shown in gold and covered by an omit map contoured at 1.0 σ. Residues forming the substrate-binding pocket are shown in silver. The red sphere represents a water molecule that is hydrogen bonded to N1.

There are two molecules per asymmetric unit in the co-crystal structure of TGEV M^pro^ with N1, compared with as many as six molecules per asymmetric unit in the native enzyme structure [37]. N1 binds to TGEV M^pro^ in a similar mode to SARS-CoV M^pro^ with some subtle differences (see Figure 3). First, after the nucleophilic addition reaction, the Michael acceptor does not remain in a plane as in the SARS-CoV M^pro^ complex structure, but instead flips over by about 90° to interact with the backbone atoms of residues 141–142. Unlike the SARS-CoV M^pro^ complexed with N1, the TGEV M^pro^ lacks a water molecule connecting the ethyl ester with the side chain of residue 142 (Asn → Ala in TGEV M^pro^). The rate of chemical inactivation presumably depends on how the reactive vinyl group is oriented and on the extent to which the transition-state intermediate can be stabilized by proteases [42]. We suspect that in SARS-CoV M^pro^, the water molecule prevents the Michael acceptor from reorienting to accept a proton from the imidazole of 41-His in the transition state. Although the intermediate remains to be unveiled, this could partially explain why N1 has a higher inactivation rate constant (*k*_3_) against TGEV M^pro^ than SARS-CoV M^pro^s. Second, another water molecule in the TGEV M^pro^ complex occupies an equivalent position to the 189-Gln side chain, which interacts with the backbone NH of Leu at P2 in SARS-CoV M^pro^–N1 complex. This water molecule, however, donates a 2.6-Å hydrogen bond to 47-Thr and accepts a 2.7-Å hydrogen bond from the NH backbone of Leu at P2. Third, the isoxazole sways to interact with the backbone atoms of residues 188–189. It is worth mentioning that these slight variations do not notably affect the *K_i_,* as the binding modes of P1, P2, and P4 remain the same as in SARS-CoV M^pro^.

### HCoV-229E, FIPV, and MHV Inhibition Assays

**Figure 4:**
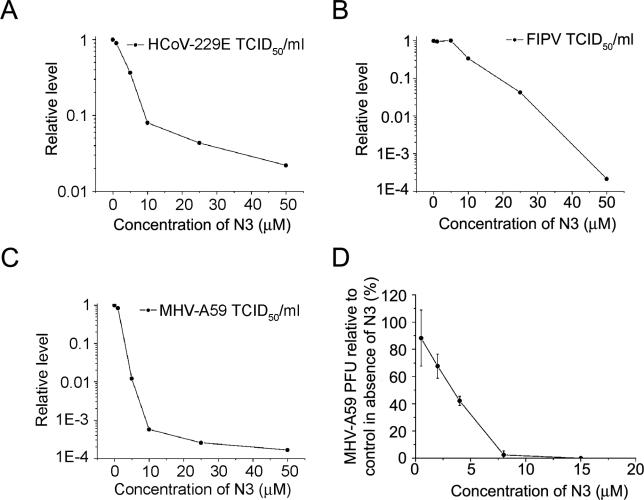
Cell-Based Assays of N3 against HCoV-229E, FIPV, and MHV-A59 Inhibition of replication of three CoVs under high-multiplicity single-cycle growth conditions (MOI = 3) and protection of DBT cells from MHV infection under low-multiplicity growth conditions (MOI = 0.01). (A) Reduction of HCoV-229E titer in MRC-5 cell culture by N3. (B) Reduction of FIPV titer in FCWF cell culture by N3. (C) Reduction of MHV-A59 titer in DBT cell culture by N3. In (A–C), infections were done at an MOI of 3 TCID_50_ per cell, and titers were determined at 14 h postinfection. (D) Plaque-reduction assay of MHV-A59. FCWF, *F. catus* whole fetus; MOI, multiplicity of infection.

Despite the high multiplicity and single-cycle infection conditions, N3 displayed potent inhibition against HCoV-229E, FIPV, and MHV-A59 with individual IC_50_ of 4.0 μM, 8.8 μM, and 2.7 μM, respectively (see Figure 4A–4C). The dose response curves all show that N3 was able to penetrate cells derived from different species and tissues to access its targets. Consequently, the results strongly imply that N3 was a wide-spectrum anti-CoV lead compound. However, we noticed some small discrepancies in the data between enzyme-inhibition assays and cell-based assays. This can be explained by the different cells for the inhibitor to enter and by potential incongruities in the dependence of M^pro^ for different CoVs. Furthermore, we cannot exclude the potential existence of differences among the bacterially expressed proteases in enzyme-inhibition assays and subtle differences in activity that were not fully revealed by the SARS-CoV-derived substrate used in our in vitro assays.

### MHV Plaque-Reduction Assay

To further substantiate the data and, in particular, to evaluate the ability of this type of compound to prevent cells from being infected by CoVs and their cellular cytotoxicity, a murine delay brain tumor (DBT) cell-based MHV plaque-reduction assay was performed for the following reasons: (1) three important human pathogens HCoV-HKU1, HCoV-OC43, and SARS-CoV belong to or relate to group II CoVs; (2) aged mice have been successfully used as a model for increased severity of SARS in elderly humans [44]. The EC_50_ of the MHV plaque-reduction assay was 3.4 μM (see Figure 4D), which was consistent with the IC_50_ determined in the MHV inhibition assay. It was observed that when the concentration of N3 increased to 8 μM, the DBT cells could be sufficiently protected. Moreover, 500 μM N3 only displayed 28.3% inhibition of cell growth, suggesting extremely low cellular toxicity (see Figure S3). These results demonstrate that N3 is a particularly promising lead compound for further development.

### Future Prospects

Evidence suggests that CoVs may have completed at least two animal-to-human interspecies transmissions to date [22,24,25]. An alternative hypothesis has been proposed whereby the 1889–1890 pandemic characterized by malaise, fever, pronounced central nervous system symptoms, with a significant increase in case fatality with increasing age, was the result of interspecies transmission of bovine CoV to humans rather than an influenza virus [25]. Although this hypothesis needs more evidence to support, it is widely acknowledged that SARS resulted from animal-to-human transmission of a previously unknown CoV. CoVs, especially those that can infect hosts such as domestic animals and pets, which humans have frequent contact with, remain a potential threat to human health assuming they cross the interspecies barrier again. Hence, the development of wide-spectrum drugs will lead to increased protection of human health, a reduction of the considerable economic costs associated with CoVs, defense against endangered wild animals susceptible to infection, and valuable model animals such as transgenic mice with high mortality rates for CoVs. Identification of the CoV M^pro^ as a conserved target among all CoVs will provide an opportunity for the development of broad-spectrum inhibitors against all CoV-related diseases. Ruprintrivir, whose backbone was also a *trans*-α, β-unsaturated ester incorporated with the peptidyl portion, has entered clinical trials against rhinovirus infection [42], although it did not show inhibition of CoVs [20]. This is a compound with poor aqueous solubility and low oral bioavailability in animals. In preclinical animal studies, hydrolysis of this compound produced alcohol and carboxylic acid metabolite, which was 400-fold less active than ruprintrivir and was the predominant biotransformation pathway. Ruprintrivir is formulated as a suspension for intranasal delivery. Phase II studies reported ruprintrivir prophylaxis reduced the proportion of subjects with positive viral cultures and viral titers. Ruprintrivir is well tolerated, and the most common adverse effects of this compound are blood-tinged mucus and nasal passage irritation [45,46]. This highlights that structure-assisted optimization of N3 could possibly lead to the discovery of a single agent to enter clinical trials against all CoV-associated diseases, although ultimate clinical potential requires more sufficient investigation. Our latest results show that N3 could also strongly inhibit the replication of SARS-CoV and TGEV in cell-based assays (data to be published elsewhere). Furthermore, since this compound was designed against a highly conserved region within the genus *Coronavirus,* it should have efficient resistance to the high mutation and recombination rates of CoVs. It is noteworthy that N3 also exhibited potent inhibition on the M^pro^s of HCoV-NL63 and HCoV-HKU1, two recently identified HCoVs associated with bronchiolitis, conjunctivitis, and pneumonia [2,3], in preliminary inhibition assays (see Table S2). This strongly supports our hypothesis that a single agent developed from N3 could provide an effective first line of defense against future emerging CoV-related diseases. Moreover, it also suggests that incorporation of Michael acceptor with the peptidyl portion specific for proteases would be a good starting point for the development of inhibitors against viral Cys or Ser proteases. A comprehensive and systematic program of optimization of this class of inhibitors based on CoV M^pro^-inhibitor complexes is underway. We have so far crystallized MHV M^pro^, and the crystallization of M^pro^s of recently identified HCoV-NL63 and HCoV-HKU1 are in progress.

## Materials and Methods

### Protein cloning, expression, and purification

The preparation of SARS-CoV M^pro^ for structural analysis has been described previously [38]. The method of preparation of SARS-CoV M^pro^ for activity assay is almost identical except that the coding sequence was inserted into BamHI and XhoI sites of the expression vector pGEX-4T-1 (Pharmacia, New York, United States). The cDNA encoding IBV M^pro^ (M41 strain) was a gift from Professor Ming Liao (South China Agricultural University, China); the cDNA encoding M^pro^ of MHV (A59 strain) was a gift from Professor Guangxia Gao (Institute of Biophysics Chinese Academy of Sciences, China); the cDNA encoding M^pro^ of HCoV-HKU1 was kindly provided by Professor Kwok-yung Yuen (University of Hong Kong, China); coding sequences of TGEV, IBV, HCoV-HKU1, and HCoV-NL63 M^pro^s were inserted into BamHI and XhoI sites of the pGEX-4T-1 plasmid, and the subsequent methods for expression and purification were carried out as for SARS-CoV M^pro^. After change of a BamHI cleavage site at 429–434 in the sequence coding MHV M^pro^ to GGCTCC, this coding sequence was inserted into BamHI and XhoI sites of pGEX-4T-1 plasmid for expression. FIPV M^pro^ (15 mg/ml) and HCoV-229E M^pro^ (15 mg/ml; two amino acids deleted at C-terminal) were expressed and purified as described previously [39,47].

### Crystallization and data collection

SARS-CoV M^pro^ was crystallized as previously reported [38]. The SARS-CoV M^pro^ inhibitor complexes were prepared as follows. First, the inhibitors were dissolved in 7.5% PEG 6000, 6% DMSO, and 0.1 M Mes (pH 6.0) with a concentration of 10 mM (supersaturation). Then, a 3-μl aliquot of such solution was added to the drop, and the crystals were soaked for approximately 2–6 days. A single crystal was prepared for low-temperature data collection by transfer to a cryoprotectant solution containing 30% PEG 400 and 0.1 M Mes (pH 6.0) and then flash frozen in a stream of N_2_ gas at 100 K. The set of SARS-CoV M^pro^-I2 complex data was collected to 2.7 Å resolution using a Mar345 image plate (Marresearch, Norderstedt, Germany) mounted on a Rigaku RU2000 X-ray generator (Sevenoaks, United Kingdom) operated at 48 kV and 98 mA (λ = 1.5418 Å). Data for SARS-CoV M^pro^ individually complexed with N1 and N3 were collected at 100 K in-house on a Rigaku CuK_α_ rotating-anode X-ray generator (MM007) at 40 kV and 20 mA (λ = 1.5418 Å) with a Rigaku image-plate detector. Data for SARS-CoV M^pro^-N9 complex were collected at 100 K in-house on a Rigaku CuK_α_ rotating-anode X-ray generator (FR-E) at 45 kV and 45 mA (λ = 1.5418 Å) with a Rigaku image-plate detector.

In respect to TGEV M^pro^ co-crystal preparation, TGEV M^pro^ was incubated with a 3-fold molar excess of N1 for 24 h at 4 °C. Crystallization trials were performed by the method published previously [37]. Briefly, the condition for crystal growth is 0.1 M HEPES (pH 8.5), 1.8 M (NH_4_)_2_SO_4_, 6% MPD, 5 mM DTT, and 5% dioxane. The set of TGEV M^pro^-N1 complex data was collected according to the method for SARS-CoV M^pro^-N9 complex All intensity data were indexed, integrated, and scaled with the HKL2000 programs DENZO and SCALEPACK [48]. Data collection statistics are summarized in Table S1. Since the refinement of the IBV M^pro^ structure is ongoing, the methods of crystallization and structure determination will be published elsewhere.

### Structure elucidation, model building, and refinement

The methods for structure determination, model building, and refinement were published previously [38]. Briefly, the SARS-CoV M^pro^-I2 complex structure was determined by molecular replacement from our native structure of SARS-CoV M^pro^ (pH 7.6) (PDB ID: 1UK3). The structures of SARS-CoV M^pro^ in complex with N1, N3, or N9 were determined from the isomorphous SARS-CoV M^pro^-I2 complex structure. The TGEV M^pro^-N1 structure was determined by molecular replacement using a single monomer of the native TGEV M^pro^ structure (PDB ID: 1P9U). All cross-rotation and translation searches for molecular replacement were performed with CNS [49]. Adjustments to the models were made in O [50]. Positional refinement, individual B-factor refinement, and water picking were performed with CNS [49]. Validation of the final models was performed with PROCHECK [51]. Detailed refinement statistics are summarized in Table S1.

### Enzyme activity assay

The activity of M^pro^s was measured by continuous kinetic assays, using an identical fluorogenic substrate MCA-AVLQSGFR-Lys(Dnp)-Lys-NH2 (over 95% purity, GL Biochem Shanghai Ltd, Shanghai, China). The fluorescence intensity was monitored with a Fluoroskan Ascent instrument (ThermoLabsystems, Helsinki, Finland) using wavelengths of 320 and 405 nm for excitation and emission, respectively. The experiments were performed with a buffer consisting of 50 mM Tris-HCl (pH 7.3), 1 mM EDTA, with or without DTT. Kinetic parameters, *K_m_* and *k_cat_,* were determined by initial rate measurements at 30 °C. With respect to SARS-CoV M^pro^, the reaction was initiated by adding protease (final concentration of 1 μM) to a solution containing different final concentrations of the substrate (3.2–40 μM). The concentrations of other M^pro^s and individual substrate range for activity assay are as follows: IBV M^pro^: 0.8 μM, substrate range: 6.4–80 μM; HCoV-229E M^pro^: 0.1 μM, substrate range: 1.6–20 μM; TGEV M^pro^: 0.1 μM, substrate range: 6.4–80 μM; FIPV M^pro^: 0.1 μM, substrate range: 1.6–20 μM; MHV M^pro^: 1 μM, substrate range: 6.4–80 μM. Fluorescence was monitored at 1 point per 2 s. Initial rates were calculated by fitting the linear portion of the curves (the first 3 min of the progress curves) to a straight line using the program Origin 7.0 (OriginLab Corporation, Natick, Massachusetts, United States). The initial velocities were converted to enzyme activity (micromole substrate cleaved)/second. Kinetic constants were obtained from a double-reciprocal plot.

### M^pro^ inhibition assays

As compounds with potent inhibition identified in preliminary inhibition assay, the strict kinetic parameters were determined. Time-dependent inhibitor progress curves were fit to a first-order exponential (equation 2) [43,52] to yield an observed first-order inhibition rate constant *(k_obs_).* P is the product fluorescence; *v*_0_ is the initial velocity; *t* is time; *D* is a displacement term to account for the fact that the emission is nonzero at the start of data collection. The values of *K_i_* and *k*_3_ were calculated from plots of 1/*k_obs_* obtained from equation 2 versus 1/[I] according to equation 3. [I] is inhibitor concentration; [S] is substrate concentration; *K_m_* is the Michaelis-Menten constant for the substrate; *k*_3_ is the rate constant of inactivation, and *K_i_* is the equilibrium constant.pt?>



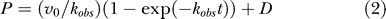





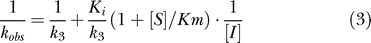



In the experiment, the *K_i_* and *k*_3_ values for the irreversible inhibitors were obtained from reactions initiated by addition of individual M^pro^, the concentration of which was similar as that for the enzymatic activity assay, containing 10 or 20 μM substrate, which depends on the enzymatic activity. The inhibitors vary from 5–8 different concentrations (10-fold molar excess of the enzyme in most cases). Data from the continuous assays were analyzed with the nonlinear regression analysis program Origin. When fast inactivation occurs, the measurement of *K_i_* and *k*_3_ proved difficult. In this case, *k_obs_*/[I] was used as an approximation of the pseudo second-order rate constant to evaluate the inhibitors and was measured at approximately 2–4 different inhibitor concentrations. The error associated with this determination (*k_obs_*/[I]) is less than 20% of a given value.

### MHV-A59 plaque-reduction assay

Murine DBT cells (generously provided by Dr. Lishan Su of University of North Carolina) were cultured in Dulbecco's modified Eagle's medium supplemented with 10% fetal bovine serum (FBS) and antibiotics at 37 °C in 5% CO_2_.

DBT cells were suspended in growth medium in triplicate wells in 6-well plates and preincubated with appropriate concentrations of the inhibitor. The next day, the medium was aspirated, and MHV-A59 was added to each well at a titer of 100 PFU/well. After incubation for 1 h, the virus inoculum was aspirated, and 2 ml of a media-agar overlay with appropriate concentrations of inhibitor was added to each well. The plates were further incubated for 24 h and stained with neutral red to visualize plaques.

### Cytotoxicity assay

DBT cells were suspended in growth medium in 96-well plates. The next day, appropriate concentrations of the inhibitor were added to the medium. Two days later, the relative numbers of surviving cells were measured by MTT (Sigma, St. Louis, Missouri, United States) assay in accordance with the manufacturer's instructions.

### HCoV-229E, FIPV, and MHV-A59 infection assays

Human embryonic lung fibroblast cells (MRC-5; ATCC [Manassas, Virginia, United States]: CCL 171), *Felis catus* whole fetus (macrophage) cells (FCWF, ATCC: CRL 2787), and DBT cells were cultured in minimal essential medium (MEM) supplemented with 25 mM HEPES, Glutamax I, nonessential amino acids, 10% FBS, and antibiotics at 37 °C in 5% CO_2_. Nearly confluent monolayers of MRC-5 (incubated at 33 °C following infection), FCWF, and DBT cells, which were grown in 6-well plates, were infected with HCoV-229E, FIPV (strain 79–1146), and MHV-A59, respectively, at a multiplicity of infection of 3 TCID_50_ per cell. After 60 min of virus adsorption, the virus inoculum was replaced with cell culture medium containing varying concentrations of N3 or in the absence of inhibitor. At 14 h postinfection, the virus titers in the cell culture supernatants were determined using standard procedures. All experiments were performed in triplicate and mean values were determined.

## Supporting Information

Figure S1 A Stereo View Showing N9 Bound into the Substrate-Binding Pocket of the SARS-CoV M^pro^ at 1.85 Å The N9 inhibitor is shown in gold and covered by an omit map contoured at 1.0 σ. Residues forming the substrate-binding pocket are shown in silver. Two water molecules (in red) form hydrogen bonds with N9.
(425 KB PDF).

Figure S2 N3 Has Wide-Spectrum Inhibition on CoV M^pro^s Activity profile curves were displayed at two different inhibitor concentrations for (A–F). (A) 0.1 μM HCoV 229E M^pro^ solution with 10 μM substrate.
(B) 0.1 μM TGEV M^pro^ solution with 20 μM substrate.
(C) 0.05 μM FIPV M^pro^ solution with 10 μM substrate.
(D) 0.6 μM MHV M^pro^ solution with 20 μM substrate.
(E) 0.8 μM IBV M^pro^ solution with 20 μM substrate.
(F) 1 μM SARS-CoV M^pro^ solution with 20 μM substrate.
(G) The preliminary inhibitory assay of N3 on M^pro^ of a newly identified CoV (HCoV-HKU1). Curve A represents the activity curve of 1 μM M^pro^ of HCoV-HKU1 in cleaving 20 μM substrate with time; curves B and C individually represent the decrease in enzyme activity when N3 was added with 2-fold and 4-fold molar of protease.
(H) The preliminary inhibitory assay of N3 on M^pro^ of a recently identified CoV (HCoV-NL63). Curve A represents the activity curve of 0.5 μM M^pro^ of HCoV-NL63 in cleaving 10 μM substrate with time; curves B and C individually represent the decrease in enzyme activity when N3 was added with 2-fold and 4-fold molar of protease.
(1.2 MB PDF).

Figure S3 The Cytotoxicity of N3 on Murine DBT Cells (124 KB PDF).

Table S1 Data Collection and Refinement Statistics (106 KB PDF).

Table S2 Representative Inhibitors Designed in the First Round (I2 not shown here) (128 KB PDF).

Table S3 Representative Inhibitors Designed in the Second Round (N1 and N3 not shown here) (121 KB PDF).

Protocol S1 (137 KB PDF).

### Accession Numbers

The Protien Data Bank (http://www.rcsb.org/pdb/) accession numbers for the structures of SARS M^pro^ individually complexed with I2, N1, N3, and, N9, and TEGV M^pro^ in complex with N1 are 1WNQ, 1WOF, 2AMQ, 2AMD, and 2AMP, respectively. The GenBank (http://www.ncbi.nih.gov/Genbank/) accession number for IBV M^pro^ is DQ157446.

## Abbreviations

CMK: chloromethyl ketone
CoV: coronavirus
DBT: delay brain tumor
DTT: dithiothreitol
FIPV: feline infectious peritonitis virus
HCoV: human coronavirus
HCoV-HKU1: HCoV strain HKU1
HCoV-NL63: HCoV strain NL63
HCoV-OC43: HCoV strain OC43
HCoV-229E: HCoV strain 229E
IBV: avian infectious bronchitis virus
MHV: murine hepatitis virus
M^pro^: main protease
SARS: severe acute respiratory syndrome
SARS-CoV: severe acute respiratory syndrome coronavirus
TGEV: porcine transmissible gastroenteritis virus
