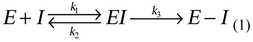# Correction: Design of Wide-Spectrum Inhibitors Targeting Coronavirus Main Proteases

**DOI:** 10.1371/journal.pbio.0030428

**Published:** 2005-11-15

**Authors:** Haitao Yang, Weiqing Xie, Xiaoyu Xue, Kailin Yang, Jing Ma, Wenxue Liang, Qi Zhao, Zhe Zhou, Duanqing Pei, John Ziebuhr, Rolf Hilgenfeld, Kwok Yung Yuen, Luet Wong, Guangxia Gao, Saijuan Chen, Zhu Chen, Dawei Ma, Mark Bartlam, Zihe Rao

In *PLoS Biology*, volume 3, issue 10, DOI: 10.1371/journal.pbio.0030324

There is a typesetting error remaining in equation 1, despite the Note Added in Proof indicating that the equation has been corrected. The second reaction step should not have a reverse arrow. The correct equation is as follows: