# Supplementary material for: Design of Wide-Spectrum Inhibitors Targeting Coronavirus Main Proteases
Source: PLoS Biol. 2005 Sep 6;3(10):e324. doi: 10.1371/journal.pbio.0030324 (PMC1197287; doi:10.1371/journal.pbio.0030324)
Supplement: Supplementary file 1 [file pbio.0030324.sd001.pdf]

Supporting on-line material

Chemical synthesis. Treatment of **1** [1] with trifluoroacetic acid followed by coupling with Boc-L-Val-L-Phg-OH **(2)** mediated by HATU afforded **I2** with 83% yield (Scheme 1). Next, the Boc protecting group in **I2** was removed, and liberated amine was coupled with 5-metylisoxale-3-carboxylic acid **(3)** to produce **I4** with 96% yield. Following similar procedures, the other six compounds in Table 2 were prepared.

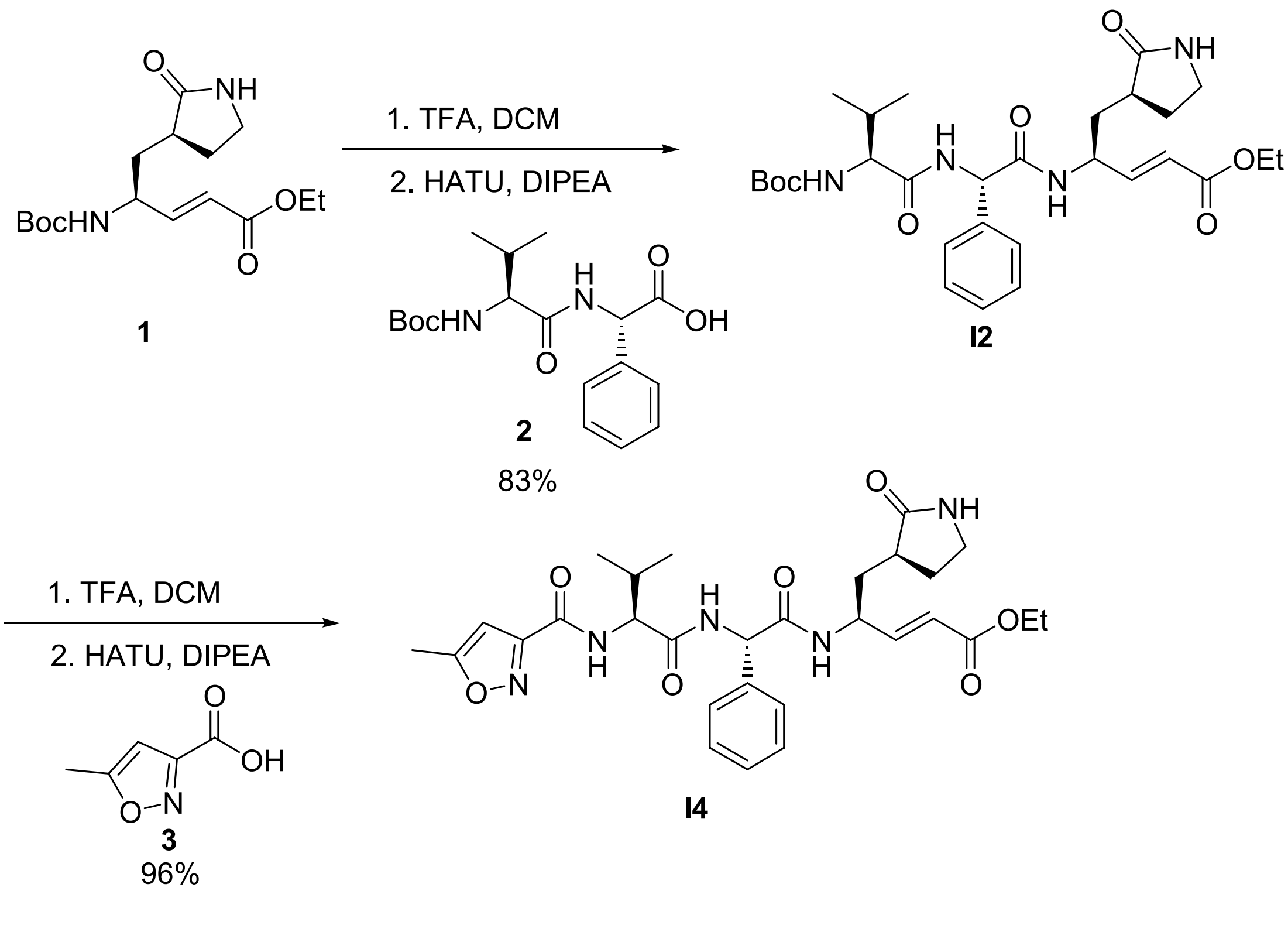

Scheme 1

The general synthesis protocol for **N3** and compounds listed in Table 3 is depicted by the preparation of **N1** and **N2** (Scheme 2). After removal of the Boc protecting group in **(1)**, coupling with Boc protected tripeptide acid **(4)** was carried out to produce **N2** with 81% yield. Switching the *N*-substituent was accompanied with treatment of **N2** with trifluoroacetic acid and coupling with 5-metylisoxale-3-carboxylic acid **(3)** to provide **N1** with 82% yield.

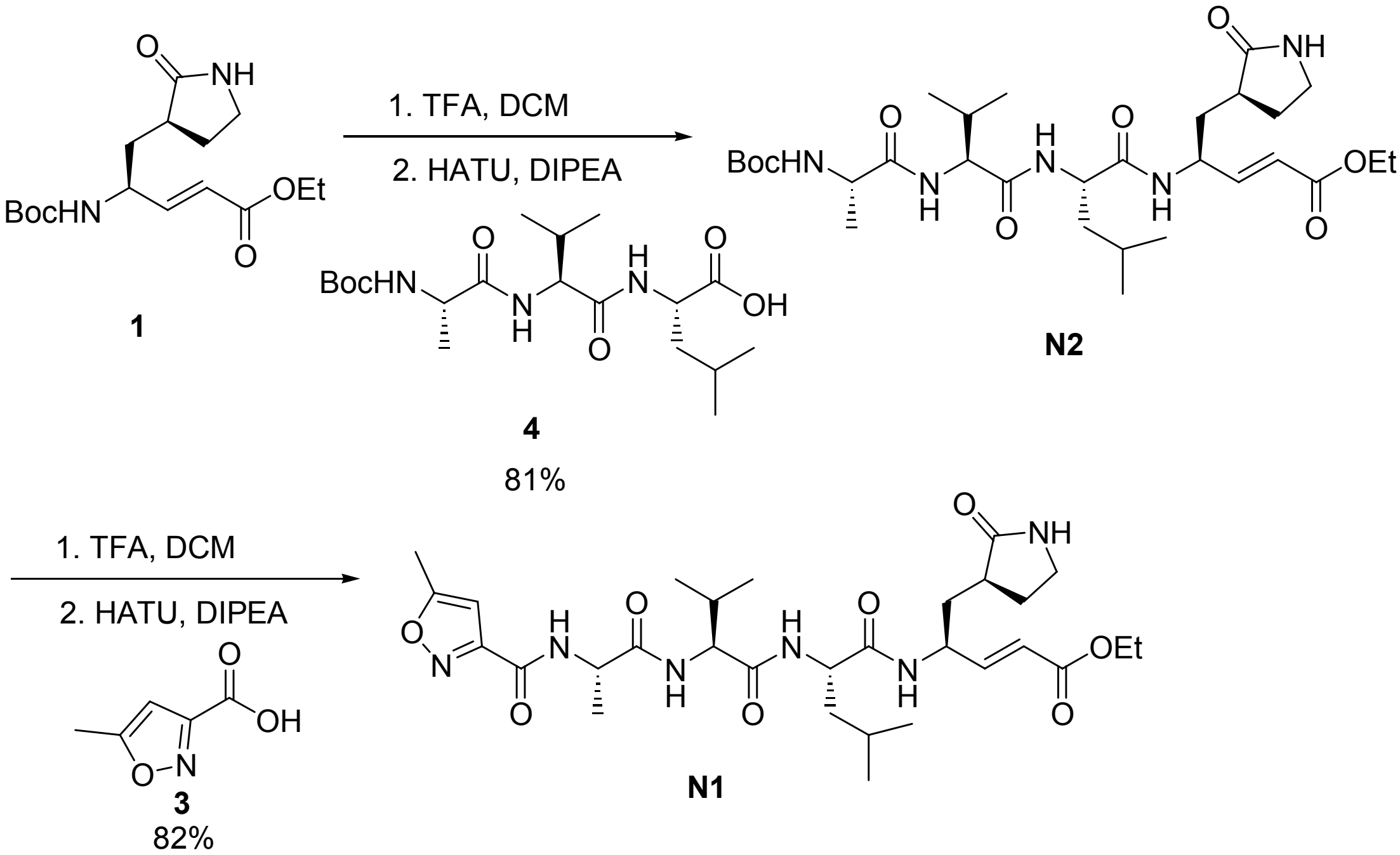

Scheme 2

Reference

1. Tian Q, Nayyar NK, Babu S, Chen L, Tao J, et al. (2001) An efficient synthesis of a key intermediate for the preparation of the rhinovirus protease inhibitor AG7088 via asymmetric dianionic cyanomethylation of N-Boc--(+)-glutamic acid dimethyl ester. Tetrahedron Letters 42: 6807-6809.
