# Supplementary material for: Design of Wide-Spectrum Inhibitors Targeting Coronavirus Main Proteases
Source: PLoS Biol. 2005 Sep 6;3(10):e324. doi: 10.1371/journal.pbio.0030324 (PMC1197287; doi:10.1371/journal.pbio.0030324)
Supplement: Supplementary file 2 [file pbio.0030324.sg001.pdf]

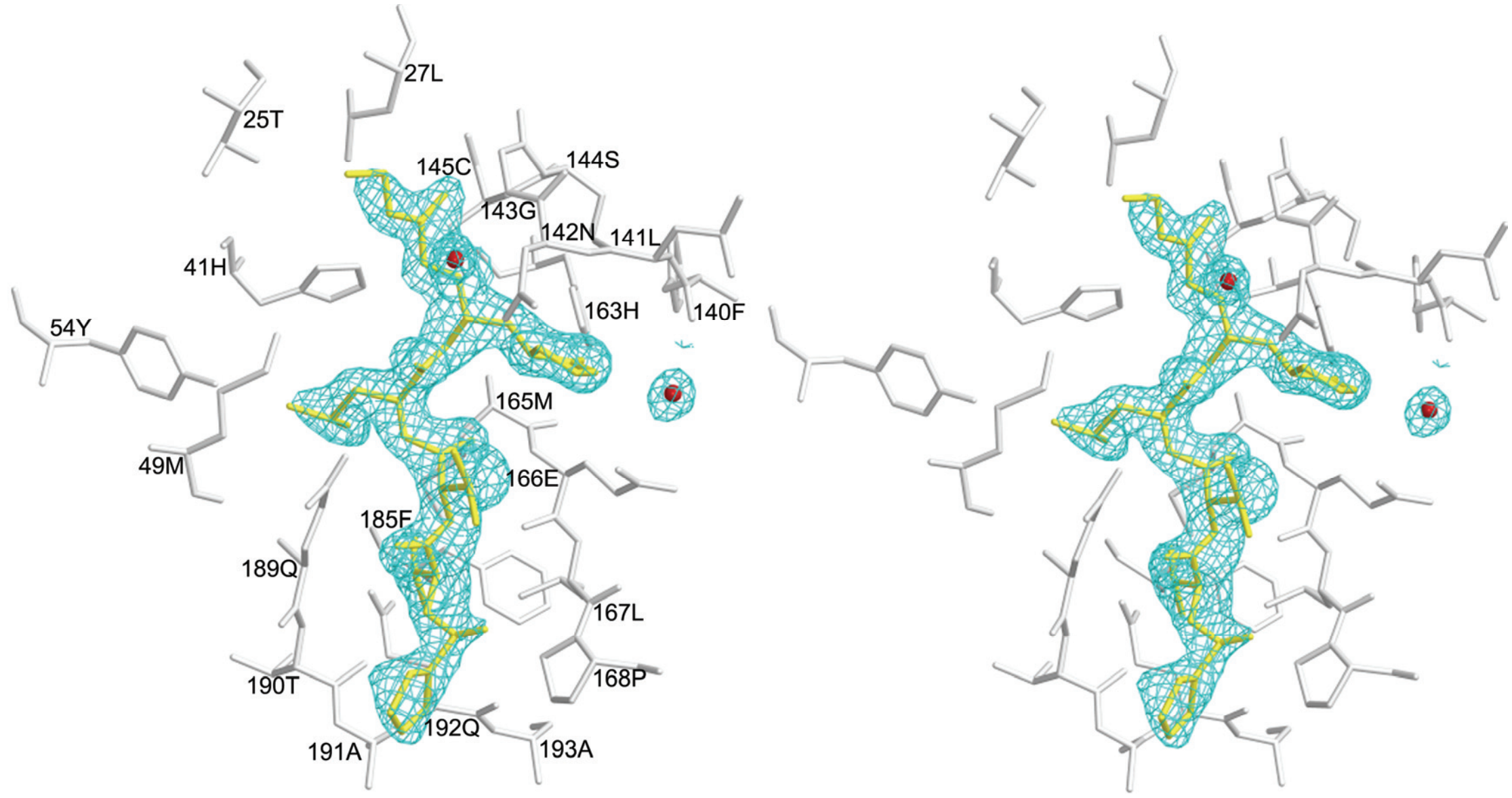

27L
25T
145C
144S
143G
142N
141L
41H
163H
140F
54Y
165M
49M
166E
185F
189Q
167L
168P
190T
192Q
191A
193A
