# Supplementary material for: Design of Wide-Spectrum Inhibitors Targeting Coronavirus Main Proteases
Source: PLoS Biol. 2005 Sep 6;3(10):e324. doi: 10.1371/journal.pbio.0030324 (PMC1197287; doi:10.1371/journal.pbio.0030324)
Supplement: Supplementary file 3 [file pbio.0030324.sg002.pdf]

A.

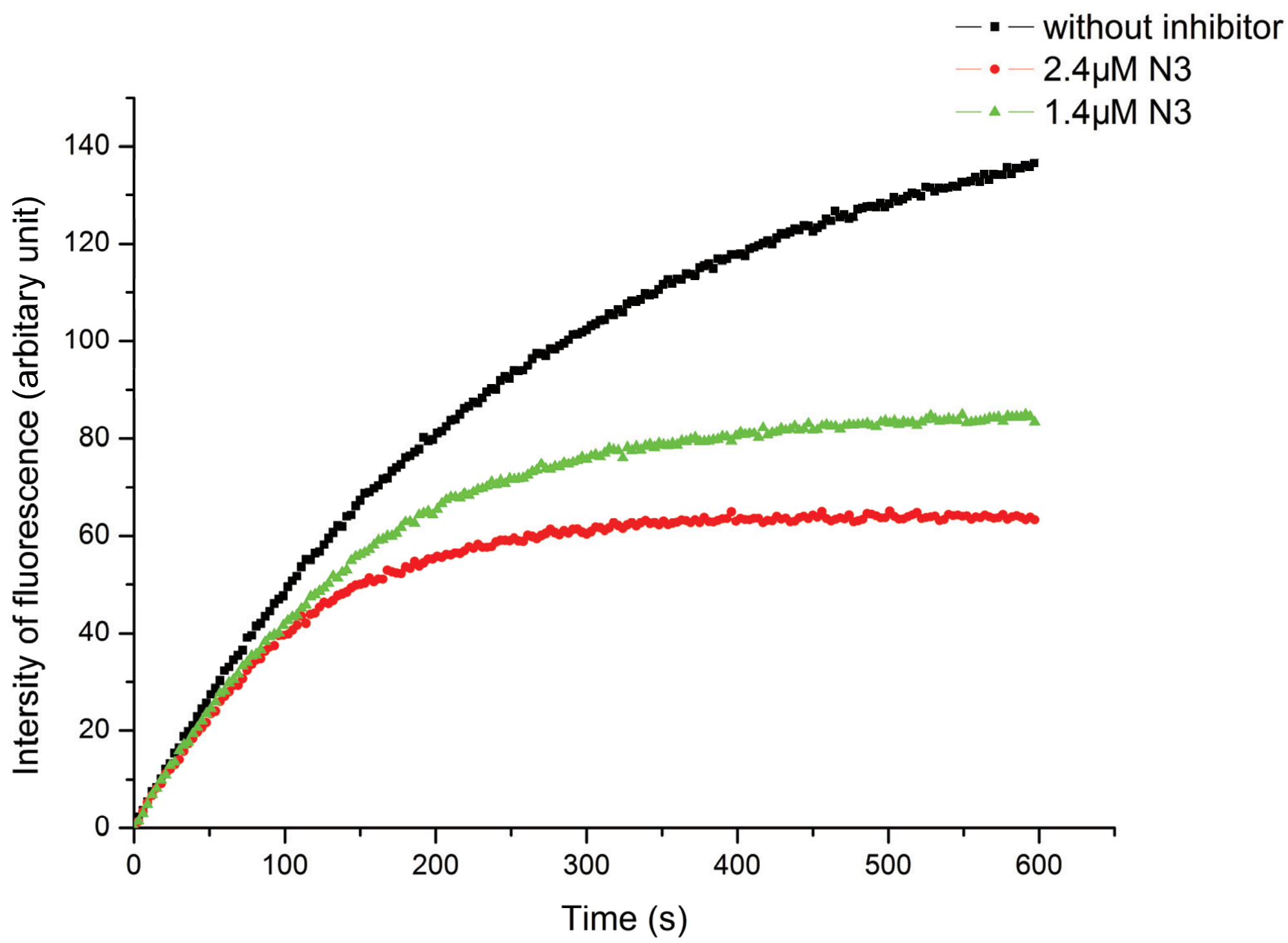
without inhibitor
2.4μM N3
1.4μM N3
Intensity of fluorescence (arbitary unit)
0
20
40
60
80
100
120
140
0
100
200
300
400
500
600
Time (s)

B.

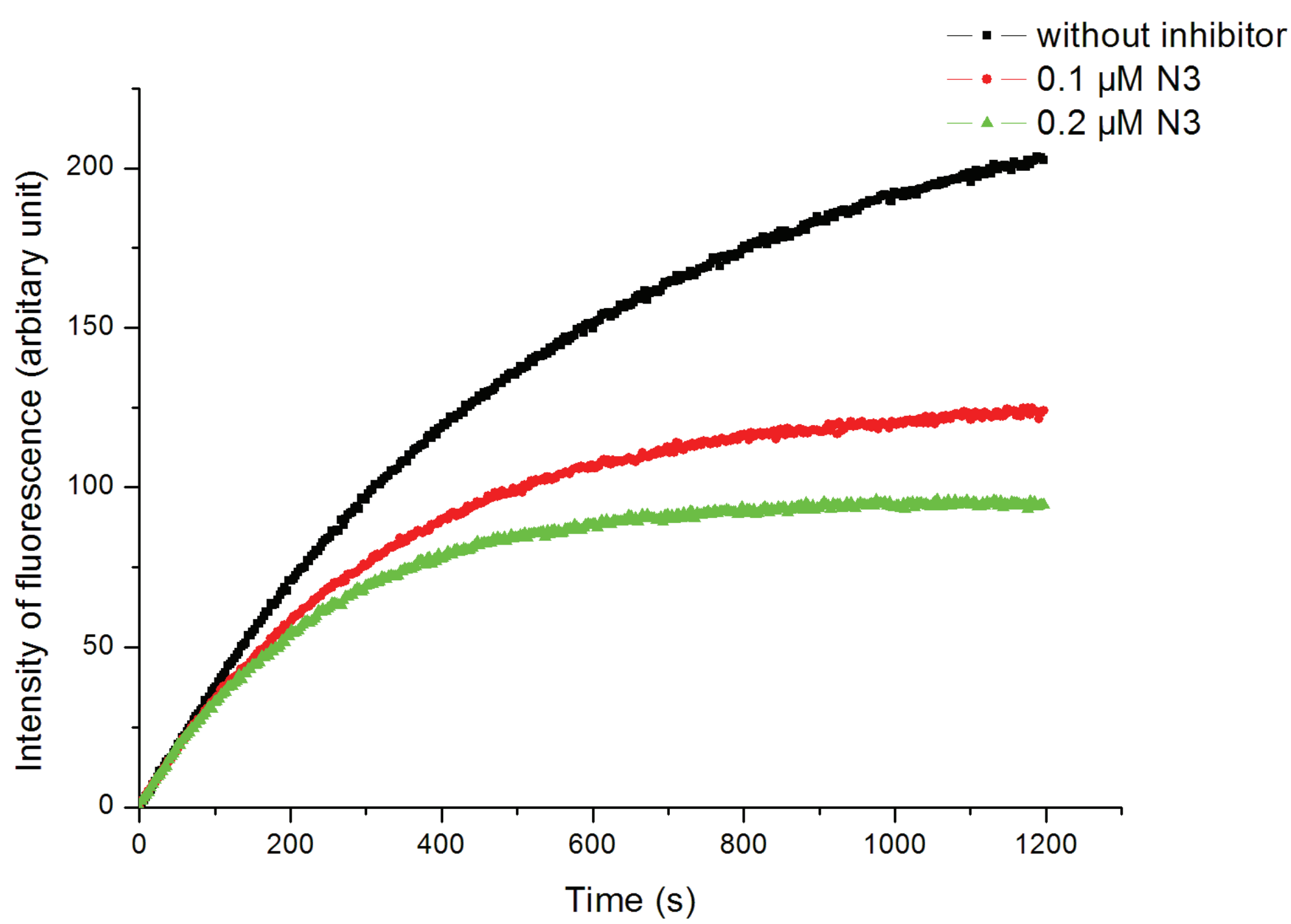
without inhibitor
0.1 μM N3
0.2 μM N3
Intensity of fluorescence (arbitary unit)
0
50
100
150
200
0
200
400
600
800
1000
1200
Time (s)

C.

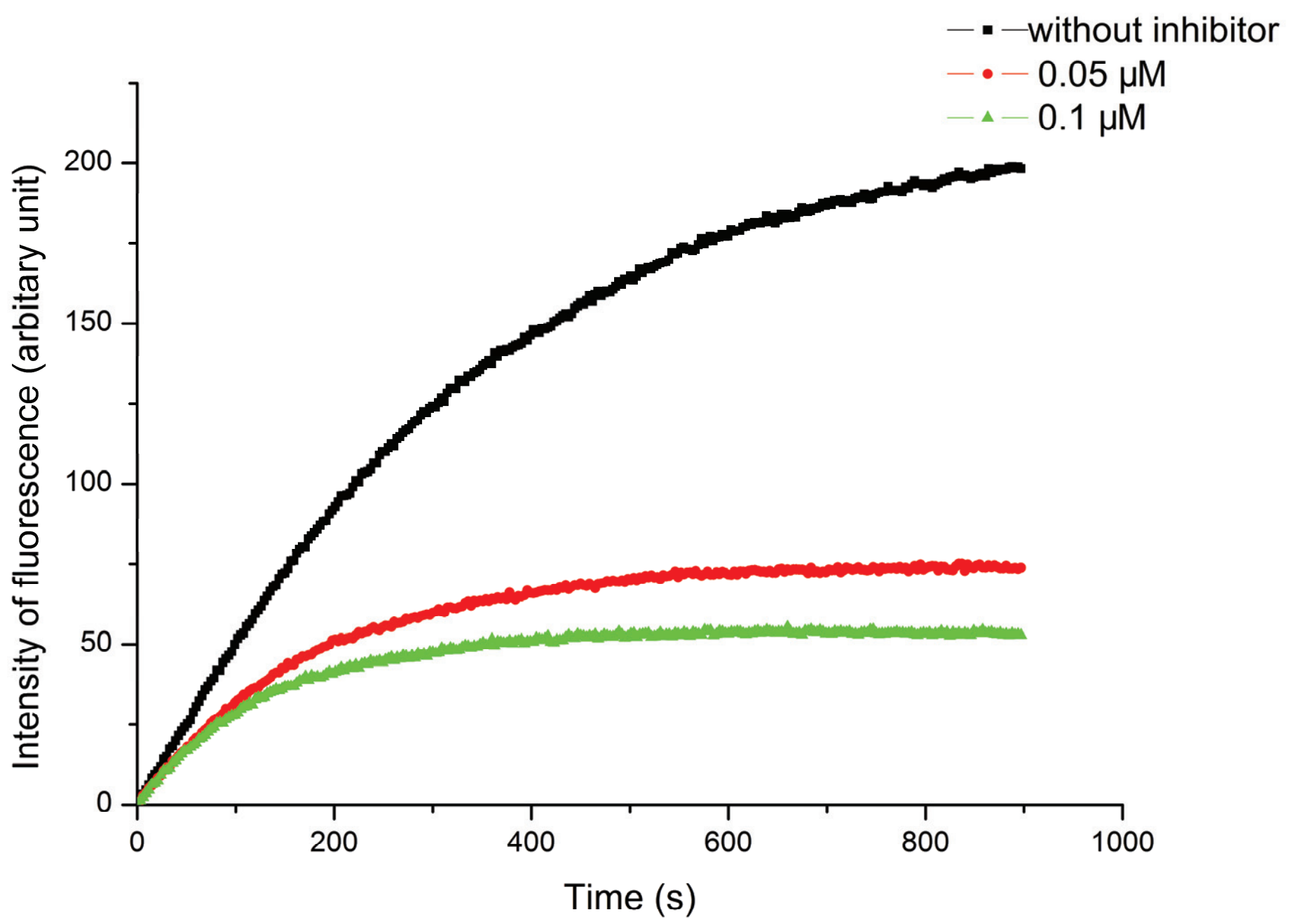
without inhibitor
0.05 μM
0.1 μM
Intensity of fluorescence (arbitary unit)
0
50
100
150
200
0
200
400
600
800
1000
Time (s)

D.

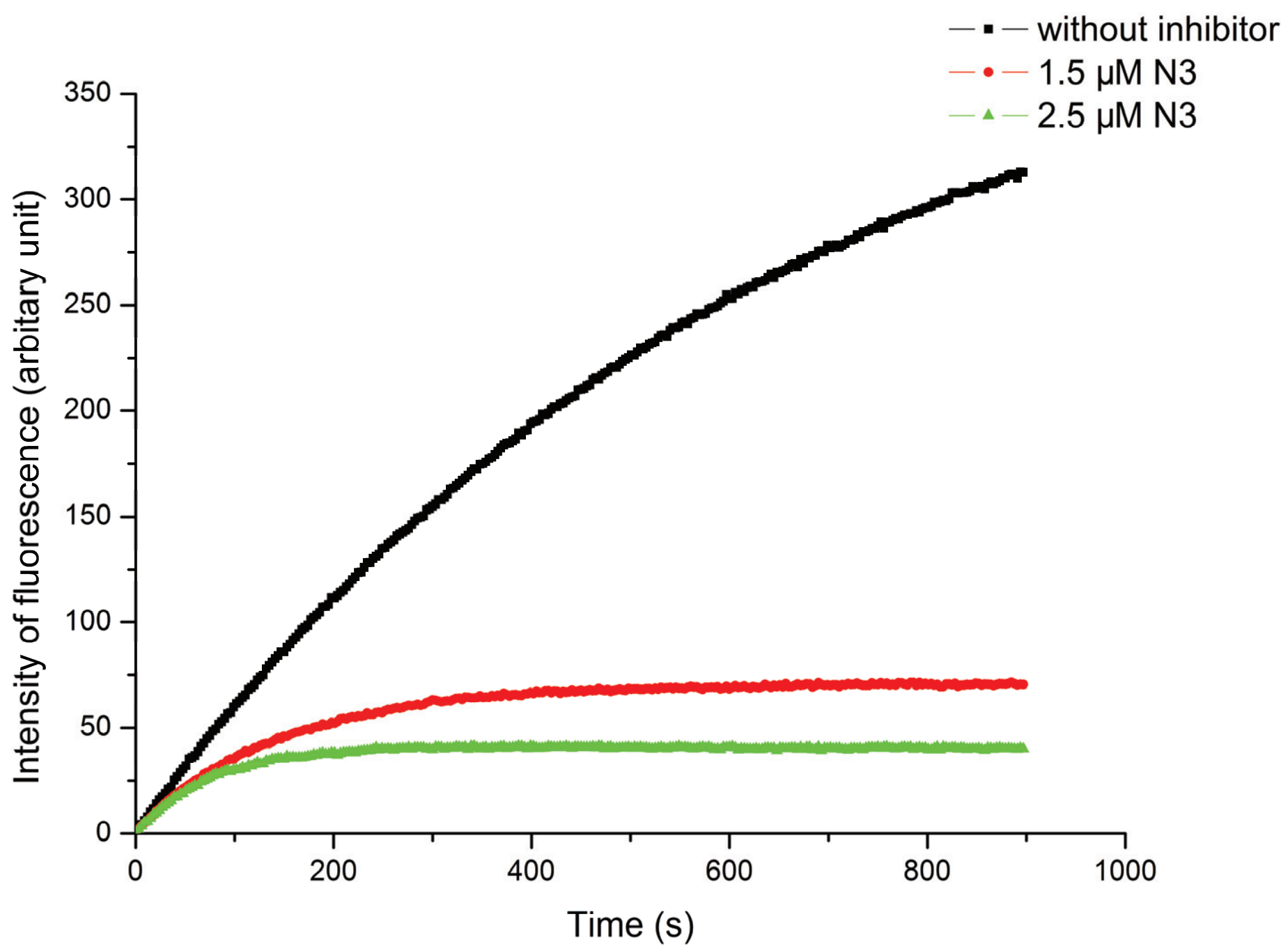
without inhibitor
1.5 μM N3
2.5 μM N3
Intensity of fluorescence (arbitary unit)
0
50
100
150
200
250
300
350
0
200
400
600
800
1000
Time (s)

E.

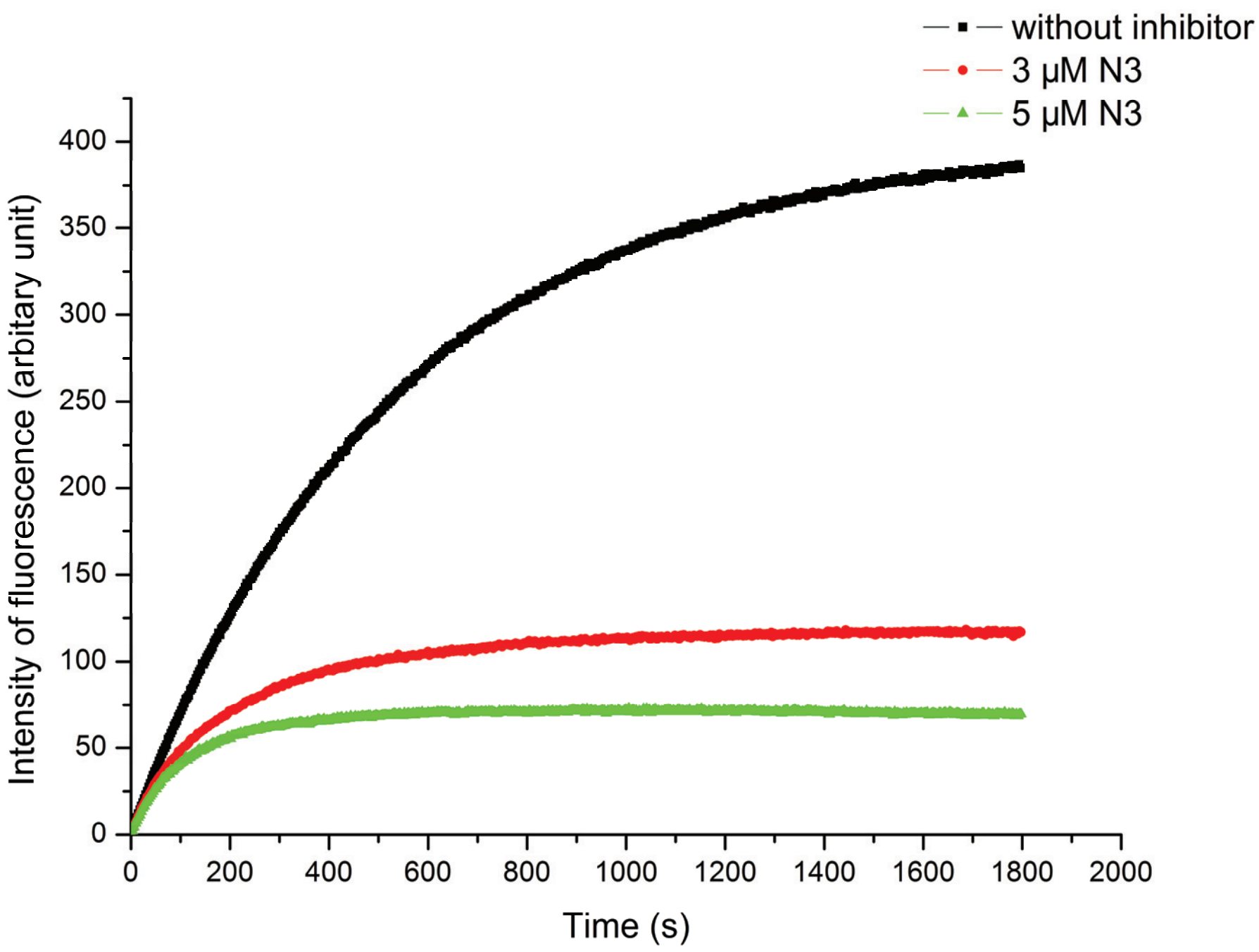

without inhibitor
3 μM N3
5 μM N3
Intensity of fluorescence (arbitary unit)
Time (s)
0
50
100
150
200
250
300
350
400
0
200
400
600
800
1000
1200
1400
1600
1800
2000

F.

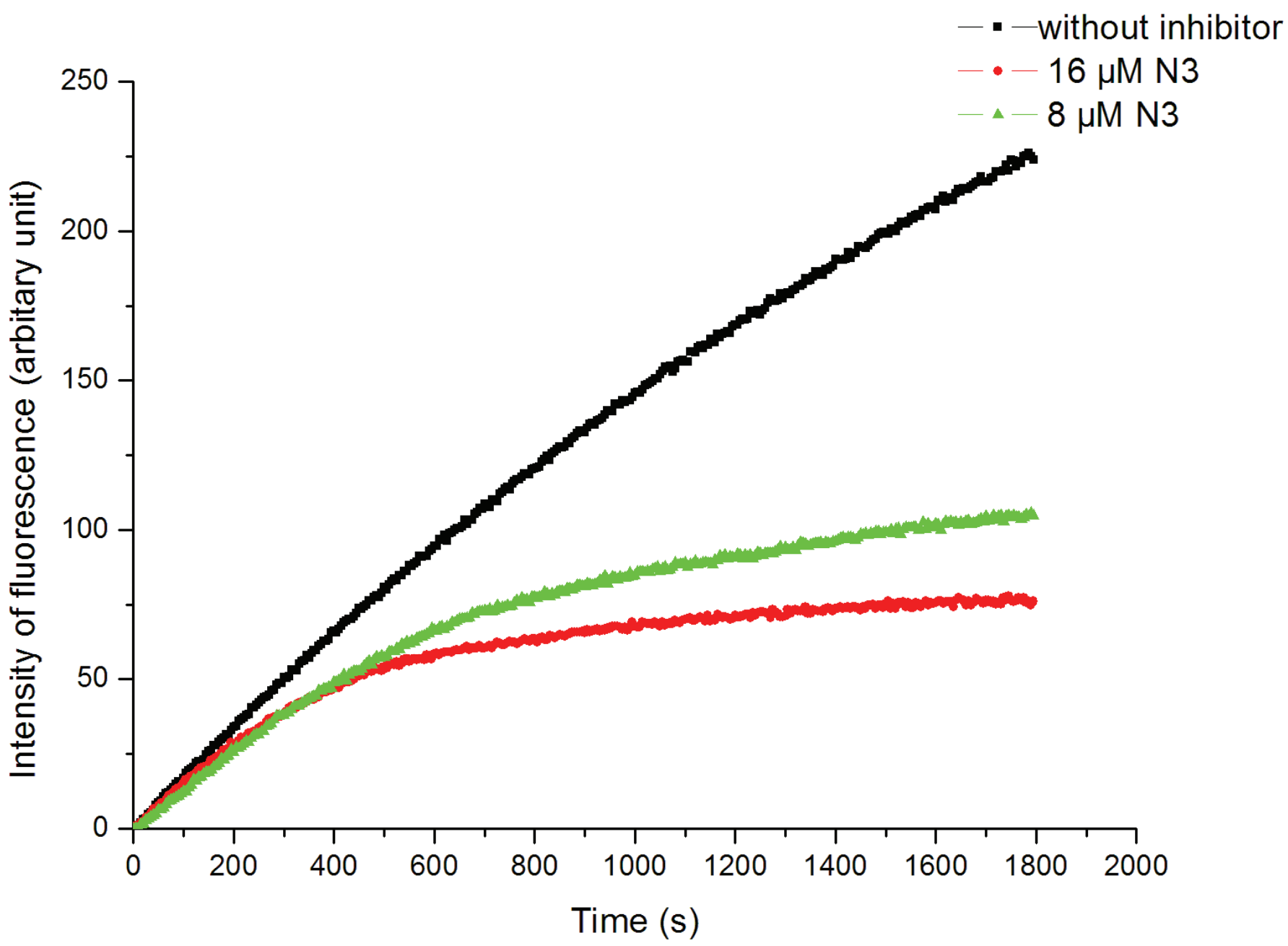

without inhibitor
16 μM N3
8 μM N3
Intensity of fluorescence (arbitary unit)
Time (s)
0
50
100
150
200
250
0
200
400
600
800
1000
1200
1400
1600
1800
2000

G.

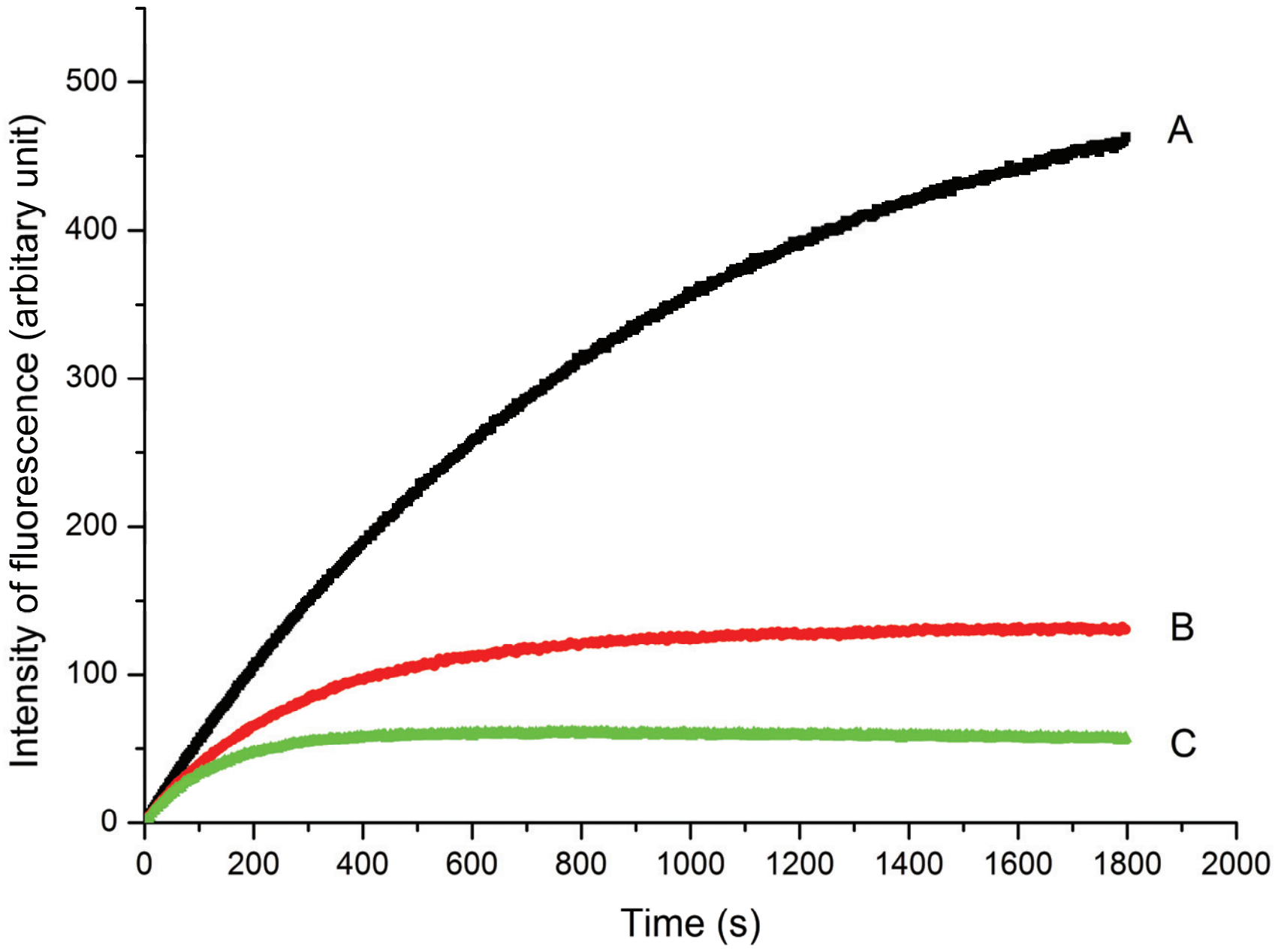

Intensity of fluorescence (arbitary unit)
Time (s)
A
B
C
0
100
200
300
400
500
0
200
400
600
800
1000
1200
1400
1600
1800
2000

H.

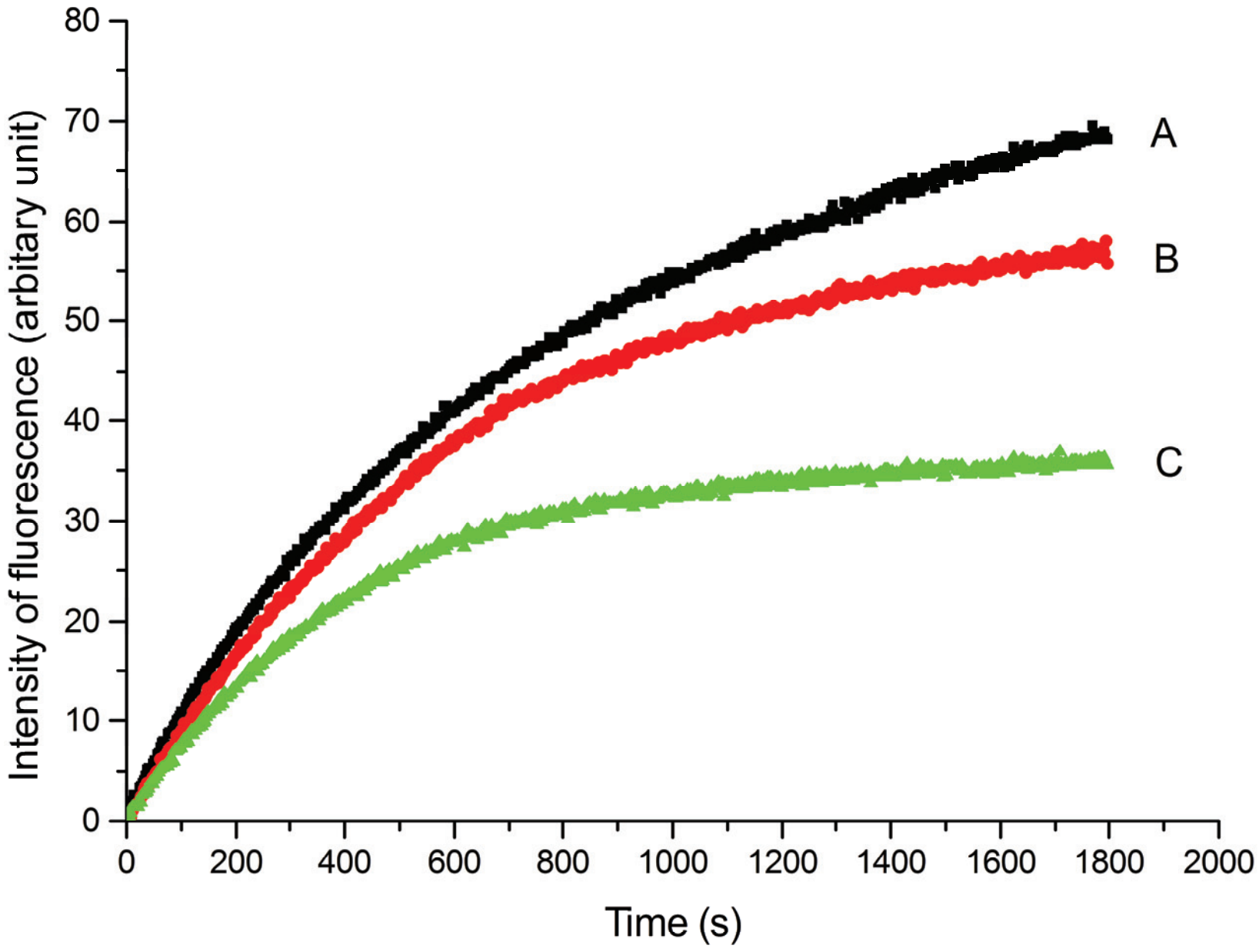

Intensity of fluorescence (arbitary unit)
Time (s)
A
B
C
0
10
20
30
40
50
60
70
80
0
200
400
600
800
1000
1200
1400
1600
1800
2000
