# Supplementary material for: Design of Wide-Spectrum Inhibitors Targeting Coronavirus Main Proteases
Source: PLoS Biol. 2005 Sep 6;3(10):e324. doi: 10.1371/journal.pbio.0030324 (PMC1197287; doi:10.1371/journal.pbio.0030324)
Supplement: Supplementary file 4 [file pbio.0030324.sg003.pdf]

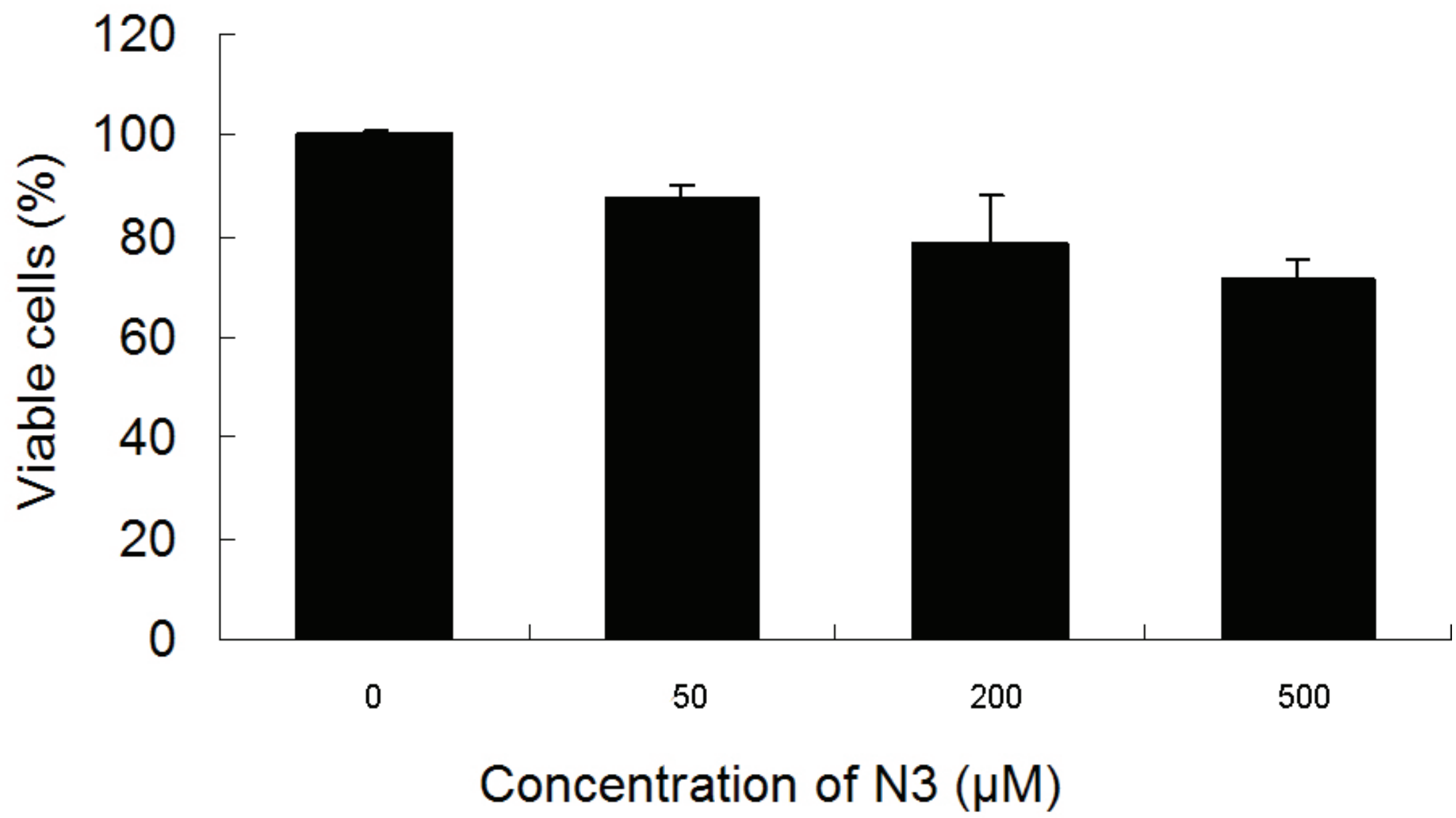

120
100
80
60
40
20
0
Viable cells (%)
0
50
200
500
Concentration of N3 (μM)
