# Supplementary material for: Design of Wide-Spectrum Inhibitors Targeting Coronavirus Main Proteases
Source: PLoS Biol. 2005 Sep 6;3(10):e324. doi: 10.1371/journal.pbio.0030324 (PMC1197287; doi:10.1371/journal.pbio.0030324)
Supplement: Supplementary file 5 [file pbio.0030324.st001.pdf]

Table 1: Data collection and refinement statistics

**Data collection statistics**					
	**SARS-CoV M^{pro}**				**TGEV M^{pro}**
	**I2**	**N1**	**N3**	**N9**	**N1**
Wavelength (Å)	1.5418	1.5418	1.5418	1.5418	1.5418
Space group	$P2_1$	$P2_1$	$P2_1$	$P2_1$	$P2_12_12_1$
Unit cell (Å, °)	a=50.7Å<br>b=94.7 Å<br>c=66.2 Å<br>β=103.0°	a=51.7 Å<br>b=95.3 Å<br>c=67.5 Å<br>β=103.6°	a=52.1 Å<br>b=95.7 Å<br>c=67.6 Å<br>β=102.9°	a=52.1 Å<br>b=95.0 Å<br>c=67.6 Å<br>β=103.1°	a=66.6 Å<br>b=90.2 Å<br>c=110.3 Å
Resolution limit (Å)	50.0 – 2.7<br>(2.8 – 2.70)	50.0 – 2.0<br>(2.08 – 2.0)	50.0 – 2.3<br>(2.34 – 2.3)	50.0 – 1.85<br>(1.93 – 1.85)	50.0 – 2.7<br>(2.75 – 2.7)
Total reflections	62,720	312,221	217,524	246,270	229,389
Unique reflections	16,799	42,325	28,372	53,796	18,149
Completeness	99.8 (99.6)	99.8 (99.7)	98.0 (96.0)	99.9 (99.5)	96.2 (92.9)
R_{merge}^{a}	9.5 (38.0)	7.8 (31.4)	12.0 (52.9)	8.3 (44.7)	12.8 (65.2)
$\langle I/\sigma(I)\rangle$	7.7 (2.5)	13.0 (3.9)	10.0 (3.3)	8.4 (2.1)	12.4 (3.8)
Redundancy	3.7 (3.7)	7.4 (7.0)	7.7 (7.7)	4.6 (4.4)	12.6 (12.8)
**Refinement statistics**					
	**I2**	**N1**	**N3**	**N9**	**N1**
Resolution range (Å)	30.0 – 2.7	30.0 – 2.0	30.0 – 2.3	30.0 – 1.85	30.0 – 2.7
R_{work}^{b} (%)	20.9	20.1	19.4	21.5	23.4
R_{free}^{b} (%)	28.2	23.9	23.0	24.5	27.1
RMS deviation:					
bonds (Å)	0.010	0.012	0.016	0.006	0.02
angles (°)	1.52	1.89	1.97	1.43	1.94
Ramachandran plot					
favored (%)	79.5	89.8	86.7	89.5	76.2
allowed (%)	18.9	9.0	12.2	9.0	21.3
generously allowed (%)	1.0	0.8	0.8	1.1	2.3
disallowed (%)	0.6	0.4	0.4	0.4	0.2

$^{a}R_{merge} = \Sigma_h\Sigma_l \mid I_{ih} - \langle I_h\rangle \mid / \Sigma_h\Sigma_I \langle I_h\rangle$, where $\langle I_h\rangle$ is the mean of the observations I_{ih} of reflection h.

$^{b}R_{work} = \Sigma (||F_p(obs)| - |F_p(calc)||)/ \Sigma|F_p (obs)|$; R_{free} = R factor for a selected subset (10%) of the reflections that was not included in prior refinement calculation.
