# Supplementary material for: Design of Wide-Spectrum Inhibitors Targeting Coronavirus Main Proteases
Source: PLoS Biol. 2005 Sep 6;3(10):e324. doi: 10.1371/journal.pbio.0030324 (PMC1197287; doi:10.1371/journal.pbio.0030324)
Supplement: Supplementary file 6 [file pbio.0030324.st002.pdf]

Table 2: Representative inhibitors designed in the first round (I2 not shown here)

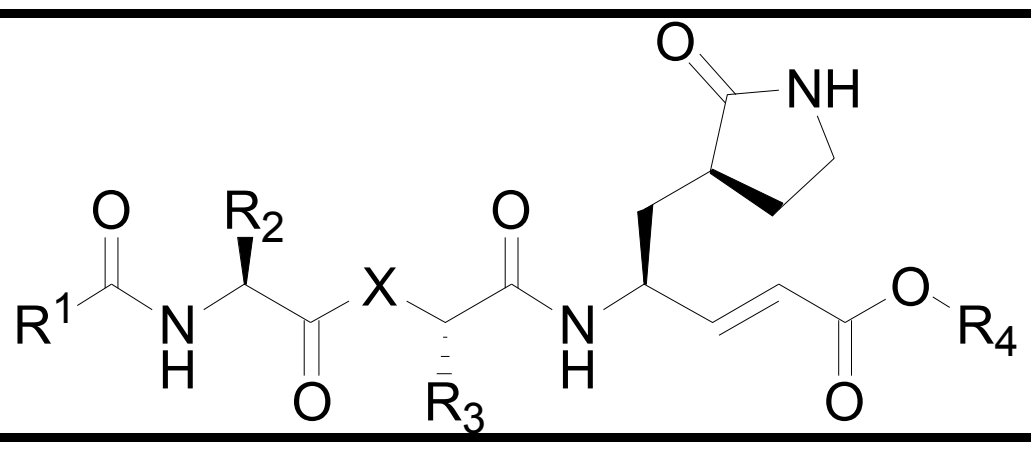

Compound No. [a]	X	R_1	R_2	R_3	R_4
**N13**	NH	Benzyloxyl	^{i}Pr	^{i}Bu	Et
**I3**	CH_2	*tert*-Butoxyl	^{i}Pr	*p*-Fluorobenzyl	Et
**ZB005**	NH	*tert*-Butoxyl	^{i}Pr	Ph	Bn
**I4**	NH	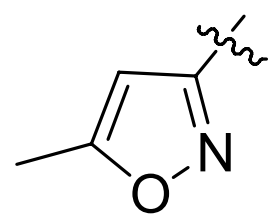	^{i}Pr	Ph	Et
**N11**	NH	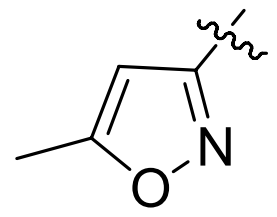	^{i}Pr	^{i}Bu	Et
**ZB006**	NH	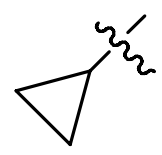	^{i}Pr	Ph	Et
**ZB007**	NH	Ph	^{i}Pr	Ph	Et

[a] Since SARS-CoV M^{pro} is the easiest to obtain, it was then used for preliminary assay of inhibitors. For the inhibitors in Table 2, even when the concentration was increased to 50 μM, there was no obvious inhibition of SARS-CoV M^{pro} after 10 minutes of observation. It suggested that these inhibitors have very poor K_i when competing with substrate.
