# Supplementary material for: Design of Wide-Spectrum Inhibitors Targeting Coronavirus Main Proteases
Source: PLoS Biol. 2005 Sep 6;3(10):e324. doi: 10.1371/journal.pbio.0030324 (PMC1197287; doi:10.1371/journal.pbio.0030324)
Supplement: Supplementary file 7 [file pbio.0030324.st003.pdf]

Table 3: Representative inhibitors designed in the second round (N1 and N3 not shown here)

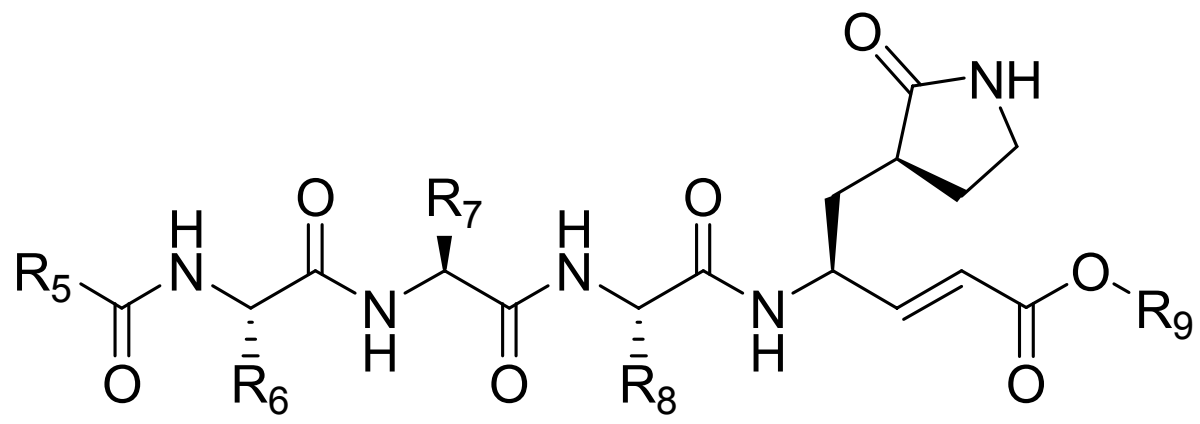

Compound No.[a]	R_5	R_6	R_7	R_8	R_9
N2	*tert*-Butoxyl	Me	^{i}Pr	^{i}Bu	Et
N4	*tert*-Butoxyl	Me	^{i}Pr	^{i}Bu	Bn
N5	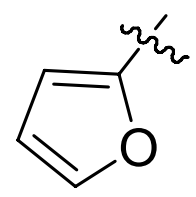	^{i}Pr	^{i}Pr	Ph	Et
N9	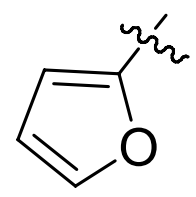	^{i}Pr	^{i}Pr	^{i}Bu	Et
N14	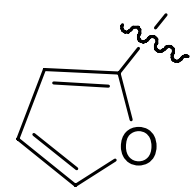	Bn	^{i}Pr	^{i}Bu	Et
N15	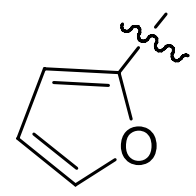	^{i}Bu	^{i}Pr	^{i}Bu	Et
N10	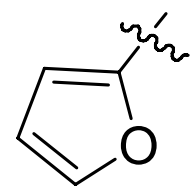	^{i}Pr	^{i}Pr	^{i}Bu	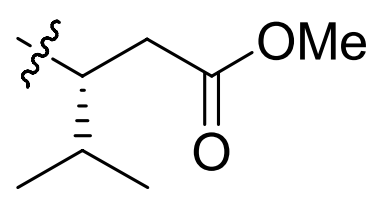

[a] In preliminary inhibition assays for SARS-CoV M^{pro}, the compounds in this table (expect N9) were less effective than N1, N3 and N9 and their accurate inhibition kinetic parameters were not determined.
